# Heterotrimetallic Au@Cu_2_Se nanozymes target inflamed neurons via suppression of oxidative stress and apoptosis to alleviate Alzheimer's disease

**DOI:** 10.1016/j.mtbio.2025.102646

**Published:** 2025-12-07

**Authors:** Chaonan Jing, Junjie Li, Dehong Yu, Minghao Chao, Hanrong Yan, Kezhen Ge, Guangyu Ma, Jiangbo Wang, Fenglei Gao, Guanqun Zhang

**Affiliations:** a Department of Neurology, Xuzhou Clinical School of Xuzhou Medical University, Xuzhou, Jiangsu, 221002, China; b Department of Neurology, Xuzhou Central Hospital, Xuzhou, Jiangsu, 221002, China; c Jiangsu Key Laboratory of New Drug Research and Clinical Pharmacy, Xuzhou Medical University, Xuzhou, Jiangsu, 221004, China; d Department of Oncology, The Affiliated Pizhou Hospital of Xuzhou Medical University, Pizhou, Jiangsu, 221399, China; e Department of Orthopaedic Surgery, Lishui Central Hospital and Fifth Affiliated Hospital of Wenzhou Medical University, Lishui, 323000, Zhejiang, China

**Keywords:** Amyloid-β, Reactive oxygen species, Metalloproteinases, Alzheimer's disease

## Abstract

Neuronal dysfunction mediated by oxidative stress and amyloid-β (Aβ) deposition is widely recognized as a core mechanism in the pathogenesis of Alzheimer's disease (AD). Aβ oligomers specifically interact with key mitochondrial proteins-such as alcohol dehydrogenase, cyclophilin D, and ATP synthase-markedly increasing reactive oxygen species (ROS) production, which leads to mitochondrial membrane potential collapse and disruption of energy metabolism. Although cuprous selenide and gold nanospheres can mimic the catalytic activities of glutathione peroxidase (GPx) and superoxide dismutase (SOD), effectively scavenge excess ROS, restore mitochondrial membrane potential, and promote ATP synthesis through synergistic action, their therapeutic potential is limited by poor targeting specificity in vivo. Moreover, while antioxidant nanoagents show promise in mitigating oxidative stress, their non-specific distribution often necessitates high doses, raising potential off-target toxicity concerns and reducing treatment efficacy. Therefore, developing a delivery system that combines multifunctional neuroprotection with precise targeting to diseased microenvironments remains an urgent need. To address this, we functionalized the surface of Au@Cs nanoparticles with hyaluronic acid (HA) to construct a CD44-targeted Au@Cs–HA–PEG nanosystem. By taking advantage of the high expression of CD44 in microglia and astrocytes under inflammatory conditions, the precise targeting of inflammatory regions in the brains of AD model mice was promoted. In vitro experiments demonstrated that Au@Cs–HA–PEG effectively reduced ROS levels in HT22 cells, reversed mitochondrial membrane potential attenuation, and restored neuronal function. In vivo results showed that these nanoparticles achieved rapid brain enrichment, significantly reduced Aβ plaque deposition and neuroinflammation, and markedly improved learning, memory, and cognitive abilities in AD mice. In conclusion, this study confirms that the Au@Cs–HA–PEG nanosystem ameliorates cognitive dysfunction in AD mice by regulating ROS homeostasis, offering a novel strategy and experimental foundation for targeted therapy of Alzheimer's disease.

## Graphical abstract

Antioxidant enzyme inhibits Aβ aggregation and the release of inflammatory factors, thereby improving neuronal function and remodeling the brain's microenvironment.

**Figure:**
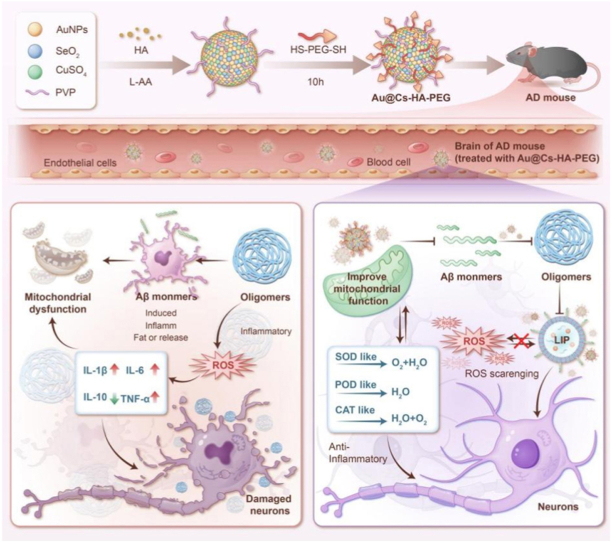

## 1 Introduction

**Scheme 1:**
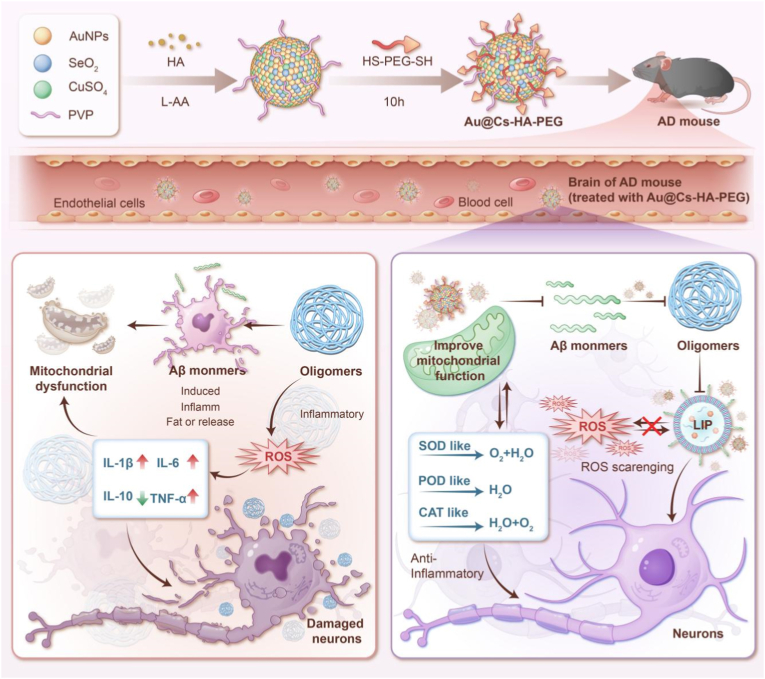
Schematic diagram of the construction process and mechanism of action of Au@Cs-HA-PEG.

Alzheimer's disease (AD) is a progressive neurodegenerative disorder of the central nervous system, primarily characterized by persistent cognitive decline and behavioral disturbances [1]. It represents the leading cause of dementia worldwide [2]. To date, elucidating the precise pathogenic mechanisms and developing effective therapeutic strategies remain major challenges in modern medicine [3]. Among the various hypotheses regarding AD pathogenesis, the theories of β-amyloid (Aβ) deposition and oxidative stress have garnered sustained attention [4,5]. Aβ can interact with multiple key proteins, markedly elevating the levels of reactive oxygen species (ROS), altering mitochondrial membrane potential, and ultimately disrupting the homeostasis of the brain's microenvironment [[6], [7], [8]]. Moreover, microglia are activated in response to Aβ deposition during the early stages of AD and attempt to clear these aggregates. As the disease progresses, however, microglial function becomes dysregulated. Not only do microglia fail to effectively clear Aβ, but they also release substantial amounts of pro-inflammatory cytokines (such as TNF-α and IL-1β), chemokines, and ROS, further exacerbating neuronal damage and synaptic dysfunction. Elevated ROS levels can form a positive feedback loop that promotes abnormal Aβ aggregation and deposition, while also polarizing microglia toward a pro-inflammatory phenotype [9,10]. This impairs their phagocytic and degradative functions, thereby perpetuating a vicious cycle of neuroinflammation and neurodegeneration [11]. However, current FDA-approved therapeutics, predominantly comprising acetylcholinesterase inhibitors and NMDA receptor antagonists, offer only symptomatic relief and do not halt the underlying pathological progression of the disease (see Scheme 1).

Oxidative stress is not merely a consequence but a core driver of Alzheimer's disease progression [12]. Aβ42 oligomers induce electron leakage from the mitochondrial electron transport chain, particularly at complexes I and III, leading to the single-electron reduction of O_2_ and generating the primary reactive oxygen species, superoxide (O_2_·^-^) [13,14]. Additionally, complexes formed by Aβ and dysregulated metal ions (such as Cu^2+^/Fe^3+^) can catalyze the oxidation of reducing agents like ascorbic acid. Importantly, sustained activation of NADPH oxidase (NOX2) in microglia results in respiratory bursts that release substantial amounts of O_2_·^-^ into the extracellular space [15,16]. The resulting O_2_·^-^ is dismutated by superoxide dismutase (SOD) or undergoes spontaneous conversion to hydrogen peroxide (H_2_O_2_), which accumulates in the AD brain due to impaired antioxidant defenses (e.g. decreased catalase and GPx activity). Through metal-dependent Fenton and Haber-Weiss reactions [17], H_2_O_2_ is further converted into highly reactive hydroxyl radicals (·OH), causing direct and irreversible damage to neuronal structures and functions. This oxidative damage further disrupts mitochondrial integrity [18], promotes metal ion release, and exacerbates neuroinflammation, forming a self-amplifying vicious cycle [19]. Therefore, inhibiting Aβ plaque formation and scavenging excess ROS represent promising therapeutic strategies for AD intervention. Cu_2_Se (Cs) nanoparticles exhibit unique enzyme-mimetic activities due to exposed Cu^+^ [20]and Se^2−^ [21] ions on their surface: they mimic glutathione peroxidase (GPx) to decompose H_2_O_2_ and lipid peroxides (LOOH) using glutathione (GSH) [22], while also modulating ROS generation via Fenton-like reactions to induce adaptive antioxidant responses [23]. Additionally, gold nanoparticles can promote the polarization of activated microglia from the pro-inflammatory M1 phenotype to the anti-inflammatory M2 phenotype, reducing the release of inflammatory factors such as tumor necrosis factor-α (TNF-α) and interleukin-1β [[24], [25], [26]] (IL-1β). This dual functionality enables precise regulation of redox homeostasis and amelioration of oxidative stress in AD. However, clinical translation of such nanoagents remains challenging due to insufficient targeting efficiency and low bioavailability in vivo [27]. Furthermore, the therapeutic efficacy of many candidate drugs targeting Aβ or tau has been hampered in clinical trials by issues such as poor blood-brain barrier (BBB) penetration, limited targeting specificity to affected brain cells, and single-target mechanisms that are inadequate against the multifactorial nature of AD. These limitations must be addressed through surface functionalization or advanced delivery systems to enhance their therapeutic potential.

Hyaluronic acid (HA), a natural polysaccharide biomacromolecule, has demonstrated considerable potential in biomedical applications, particularly in targeted delivery systems [28]. Its inherent biocompatibility, biodegradability, and affinity for specific cell receptors make it an ideal carrier for targeted therapy in inflammatory diseases. In the inflammatory microenvironment, the expression of matrix metalloproteinases (MMPs) is often markedly upregulated [29]. HA can be functionalized by incorporating enzyme-cleavable peptides to construct MMP-responsive smart drug delivery systems [30]. When such a system accumulates at inflammatory sites, locally overexpressed MMPs specifically cleave these enzyme-sensitive sequences within the HA backbone, enabling controlled drug release [31]. Moreover, effector cells such as macrophages and neutrophils activated in inflammatory regions highly express HA receptors including CD44 and RHAMM [[32], [33], [34]]. Leveraging this characteristic, HA-based nanocarriers can actively target and bind to these cells, enhancing drug accumulation at pathological sites [35]. Recent studies further suggest that exogenous supplementation of high-molecular-weight hyaluronic acid (HMW-HA) may ameliorate cognitive dysfunction through multiple mechanisms, such as restoring acetylcholinesterase activity balance, suppressing reactive astrocyte proliferation, and preserving blood-brain barrier integrity [36].

In this study, we innovatively integrated gold nanospheres, cuprous selenide, and high-molecular-weight hyaluronic acid to construct a multifunctional Au@Cu_2_Se–HA–PEG nanocomposite system capable of targeted delivery and multi-level regulation (Scheme 1). Leveraging the high affinity of hyaluronic acid for CD44 receptors—overexpressed in both peripheral and cerebral regions—the system actively targets activated microglia and reactive astrocytes, enabling precise modulation of the neuroinflammatory microenvironment [37]. The core components, comprising copper selenide and gold nanospheres, act synergistically to efficiently eliminate excess reactive oxygen species by mimicking antioxidant enzymes such as glutathione peroxidase (GPx) and superoxide dismutase (SOD) [38,39]. This activity helps reverse mitochondrial dysfunction, restore membrane potential, and promote ATP synthesis, thereby disrupting the self-amplifying vicious cycle linking Aβ aggregation and oxidative stress. This work aims to intervene at the origin of Alzheimer's disease pathological network. By remodeling intracellular redox homeostasis, attenuating neuroinflammation, and preserving synaptic integrity, the system ultimately enhances neuronal energy metabolism and improves cognitive function [40]. These findings propose a novel nanotherapeutic paradigm with translational potential to overcome the limitations of current treatment strategies.

## 2 Material and methods

### 2.1 Preparation of Au NPs

To prepare gold nanoparticles, 1.5 mL of 10 mM HAuCl_4_ solution was mixed with 100 mL of deionized water, and the resulting solution was heated to gentle boiling under condensation to prevent solvent loss. Then, 100 mL of 10 mg mL^−1^ sodium citrate solution was added to the boiling mixture under continuous stirring. The solution was maintained under boiling until a wine-red color appeared, indicating nanoparticle formation. The resulting gold nanoparticle suspension was cooled to room temperature for further use.

### 2.2 Preparation of Cu_2_Se

To synthesize Cu_2_Se nanoparticles, 16 mL of 10 mg mL^−1^ Polyvinylpyrrolidone (PVP) solution was added to 55 mL of deionized water under continuous stirring at 30 °C. Then, 1 mL of 0.1 M SeO_2_ and 3 mL of 0.2 M ascorbic acid solution were introduced. After 30 min, a mixture of 1 mL of 0.2 M CuSO_4_ and 4 mL of 0.2 M ascorbic acid was added. The reaction proceeded under stirring at 30 °C for 10 h. Finally, the resulting suspension was collected by centrifugation, washed three times with deionized water, and the Cu_2_Se nanoparticles were obtained.

### 2.3 Preparation of Au@Cs

To synthesize Au@Cu_2-x_Se nanoparticles, 11 mL of gold nanoparticle (Au NP) suspension redispersed in deionized water was mixed with 3.2 mL of 10 mg mL^−1^ PVP solution and stirred at 30 °C for 1 h. Subsequently, 0.2 mL of 100 mmol SeO_2_ and 0.6 mL of 200 mmol ascorbic acid solution were added. After 30 min, a mixture of 0.2 mL of 200 mmol CuSO_4_ and 0.8 mL of 200 mmol ascorbic acid was introduced. The reaction was allowed to proceed with stirring at 30 °C for 10 h. The resulting suspension was then collected by centrifugation, washed three times with deionized water, and the Au@Cu_2-x_Se nanoparticles were obtained.

### 2.4 Preparation of Au@Cs-HA-PEG

To synthesize Au@CS–HA–PEG nanoparticles, 4.78 mL of 1 % hyaluronic acid (HA) aqueous solution was added to 11 mL of gold nanoparticle (Au NP) solution under vigorous stirring. Then, 100 μmol of ascorbic acid and 15 μmol of selenium dioxide (SeO_2_), both in aqueous solution, were sequentially introduced into the reaction mixture. Stirring was continued until the solution turned light green. Subsequently, 30 μmol of copper sulfate (CuSO_4_) and 120 μmol of ascorbic acid in aqueous solutions were added, and stirring was maintained until a light green color reappeared. After the reaction is completed, centrifuge the mixture at 3500 rpm for 5 min. Then collect the supernatant and centrifuge it at 12,000 rpm for 10 min. Next, 0.02 g of dithiol polyethylene glycol (HS–PEG–SH, Mw = 5000) powder was added directly to the reaction flask and allowed to dissolve and react completely under magnetic stirring. Finally, the product was washed three times with deionized water to obtain the purified Au@CS–HA–PEG nanoparticle aqueous solution.

### 2.5 Characterization of Au@Cs-HA-PEG

The prepared Au@Cs-HA-PEG was characterized by transmission electron microscopy (JEM-2100PLUS) and scanning microscopy, and micrographs and elemental mapping images (JEM-F200) were obtained. X-ray diffraction (XRD) spectroscopy was determined by X-ray diffractometer (Rigaku SE, Japan). Au@Cs-HA-PEG was determined by Fourier transform infrared spectrometer to obtain FTIR spectra. The XPS spectra were obtained by X-ray photoelectron spectrometer (Thermo Fisher ESCALABXi).

## 3 Results and discussion

### 3.1 Synthesis and characterization of Au@Cs-HA-PEG

**Fig. 1:**
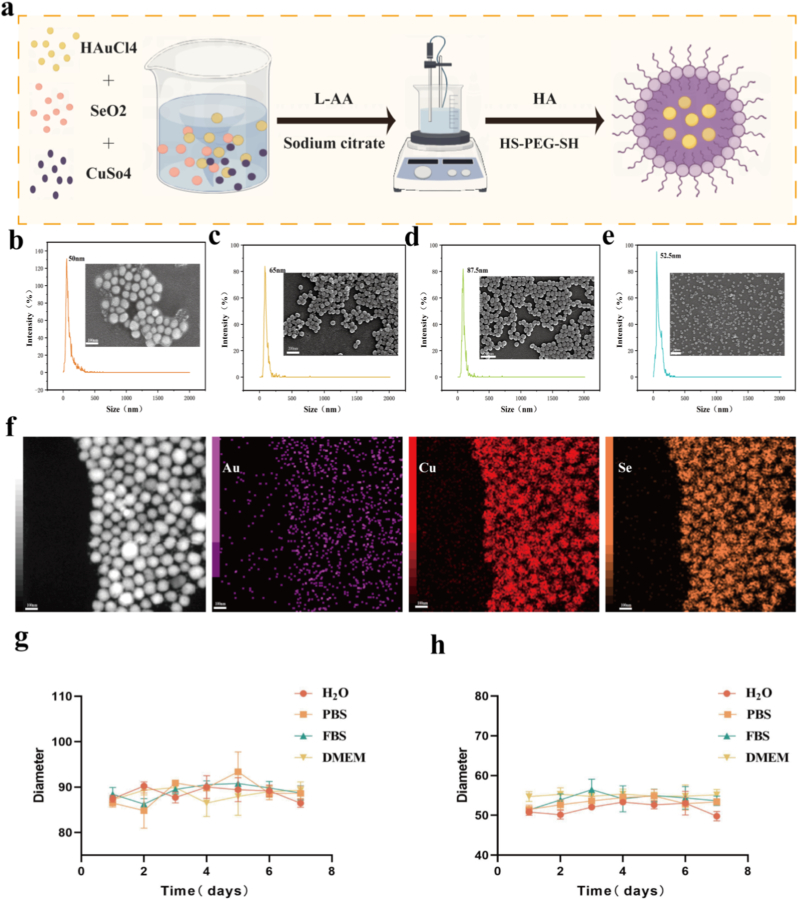
(a) Au@Cs-HA-PEG Schematic diagram of nanomaterial synthesis. (b–e) Scanning electron microscope images and particle size diagrams of Au, Cs, Au@Cs, Au@Cs-HA-PEG. (f) Mapping images of Au, Cu and Se elements of Au@Cs-HA-PEG nanoparticles. (g) Observe the hydrodynamic magnitude changes of Au@Cs in DMEM, FBS, PBS and deionized water within one week. (h) Observe the hydrodynamic magnitude changes of Au@Cs-HA-PEG in DMEM, FBS, PBS and deionized water within one week.

The synthesis of Au@Cs–HA–PEG nanoparticles is straightforward, as illustrated in Fig. 1a. Different molar ratios of tetrachloroauric acid and PVP solution were mixed, heated to 120 °C, and purified via centrifugation to obtain a gold nanosphere solution. Scanning electron microscopy (SEM) images (Fig. 1b) and dynamic light scattering (DLS) measurements confirmed the formation of gold nanospheres with an average diameter of approximately 50 nm. Subsequently, various molar amounts of selenium dioxide and anhydrous copper sulfate solutions were mixed with the gold nanosphere solution. Antioxidant ascorbic acid, hyaluronic acid, and dithiol-polyethylene glycol (HS–PEG–SH) were added, and the mixture was stirred at 30 °C for 10 h. After further purification and washing, the final product, Au@Cs–HA–PEG nanomedicine, was obtained. As shown in Fig. 1e, SEM, TEM and DLS revealed nanoparticles with an average diameter of approximately 52 nm (Fig. S1B), Additionally, SEM and DLS were used to characterize intermediate Cu_2_Se nanoparticles (Fig. 1c) and Au@Cs nanoparticles (Fig. 1d), showing uniform sizes of about 65 nm and 87.5 nm, respectively. TEM imaging of Au@Cs nanoparticles is provided in Fig. S1A. We further verified the TEM images of the nanoparticles after 7 days Fig. S2. Modification with HA and dithiol-PEG further reduced the size of the final nanomedicine, facilitating its passage across the blood–brain barrier. Element mapping confirmed the existence of Au, Cu and Se (Fig. 1f). In addition, Au@Cs and Au@Cs-HA-PEG nanoparticles demonstrated good dispersity in various solutions. We photographed and compared their states in different solutions (Fig. S3), and performed operations such as ultrasonic dispersion prior to size measurements. The results indicated that the materials maintained consistent particle sizes after 7 days of storage. As shown in Fig. 1g and h, the nanoparticles exhibit excellent stability.

**Fig. 2:**
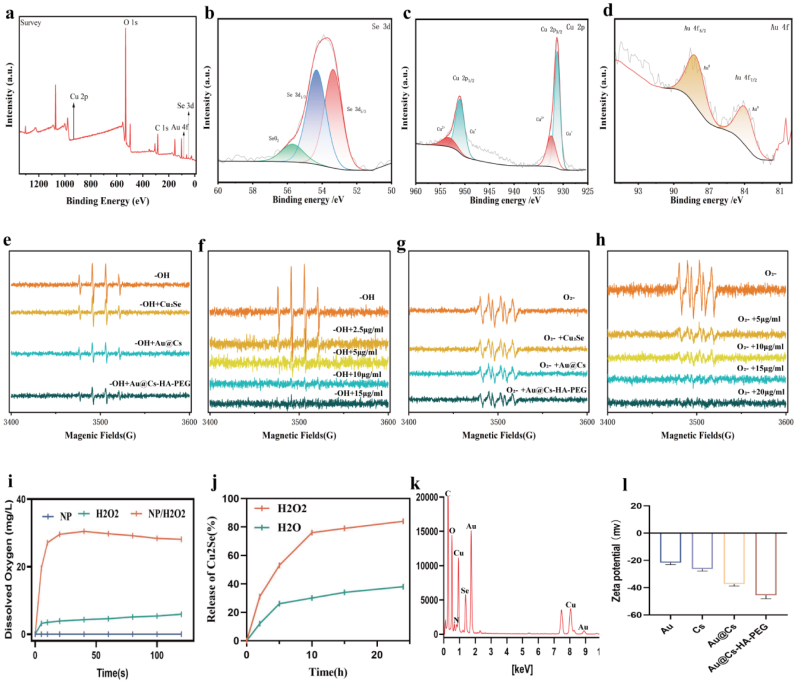
(a) XPS spectrum of Au@Cs-HA-PEG. (b) The spectrum of high-resolution xps Se. (c) The spectrum of f high-resolution xps Cu. (d) The spectrum of high-resolution xps Au. (e-h)The ESR spectra of the DMPO/•OH complex and DMPO/O_2_•− indicate the depletion of •OH and O_2_•− after incubation with different NPs. (i) Quantitative analysis of oxygen production by Au@Cs-HA-PEG NP. (j) Cs release curve of Au@Cs-HA-PEG NP. (k) EDX elemental analysis diagram of Au@Cs-HA-PEG nanoparticles. (l) ζ-Potential of Au, Cs, Au@Cs, Au@Cs-HA-PEG.

XPS analysis further verified the existence of Au, Cu, and Se in Au@Cs–HA–PEG NPs. After surface modification with HA, the zeta potential shifted significantly from −37.7 ± 0.5 mV to −45.83 ± 0.7 mV (Fig. 2l), indicating successful coating and improved colloidal stability. In the high-resolution XPS spectra, the Se 3 d region exhibited two distinct peaks at 53.11 eV and 55.02 eV, corresponding to Se 3d_5_/_2_ and Se 3d_3_/_2_, respectively, along with a minor oxidized Se peak near 56 eV. The Cu 2p spectrum showed main peaks at 930.8 eV (Cu 2p_3_/_2_) and 951.0 eV (Cu 2p_1_/_2_), with satellite features at 932.5 eV and 955.4 eV indicative of Cu^2+^. The Au 4f region displayed characteristic doublets at 84.3 eV (Au 4f_7_/_2_) and 88.3 eV (Au 4f_5_/_2_) (Fig. 2a–d). Based on this rationale, we employed electron spin resonance (ESR) spectroscopy to evaluate the antioxidant activity of Au@Cs–HA–PEG nanomedicine in scavenging hydroxyl radicals (·OH) and superoxide anions (O_2_·^-^), using DMPO (5,5-dimethyl-1-pyrroline N-oxide) as the spin trap. The radical scavenging capabilities of Au@Cs–HA–PEG nanoparticles toward ·OH and O_2_·^-^ were assessed by ESR, as shown in Fig. 2e and f. For comparison, Au, Cu_2_Se, Au@Cs, and Au@Cs–HA–PEG were analyzed under the same experimental conditions. A pronounced reduction in both DMPO/·OH and DMPO/O_2_·^-^ adduct signals was observed following treatment with Au@Cs–HA–PEG. As illustrated in Fig. 2g, the characteristic ESR signal of DMPO/·OH decreased in a concentration-dependent manner upon incubation with Au@Cs–HA–PEG, demonstrating it's potent ·OH scavenging ability. Similarly, the addition of Au@Cs–HA–PEG led to a marked decrease in the DMPO/O_2_·^-^ signal intensity, which was further suppressed at higher nanoparticle concentrations (Fig. 2h). The FT-IR spectrum showed an increased absorption peak at 3363 cm−1 corresponding to *ν*(C–H), and a peak at 1386 cm−1 attributed to the typical C–O stretching vibration, further confirming the successful synthesis of the Au@Cs–HA–PEG nanoparticles (Fig. S4). The addition of Au@Cs–HA–PEG NPs to H_2_O_2_ rapidly generates a substantial amount of O_2_, vividly demonstrating the remarkable oxygen generation capacity of Au@Cs–HA–PEG NPs (Fig. 2i). We further co-incubated Au@Cs-HA-PEG with HT22 cells and determined the levels of cuprous selenide ions in the cell culture supernatant and intracellular chambers to verify the ion sustained-release ability of Au@Cs-HA-PEG (Fig. 2j). These results confirm that Au@Cs–HA–PEG nanoparticles exhibit significant superoxide anion scavenging activity. The survey spectrum (Fig. 2k) further confirmed the presence of Au, Cu, Se, C, N, and O.

### 3.2 Investigation of the collaborative scavenging of reactive oxygen species by Au@Cs–HA–PEG and CAT

**Fig. 3:**
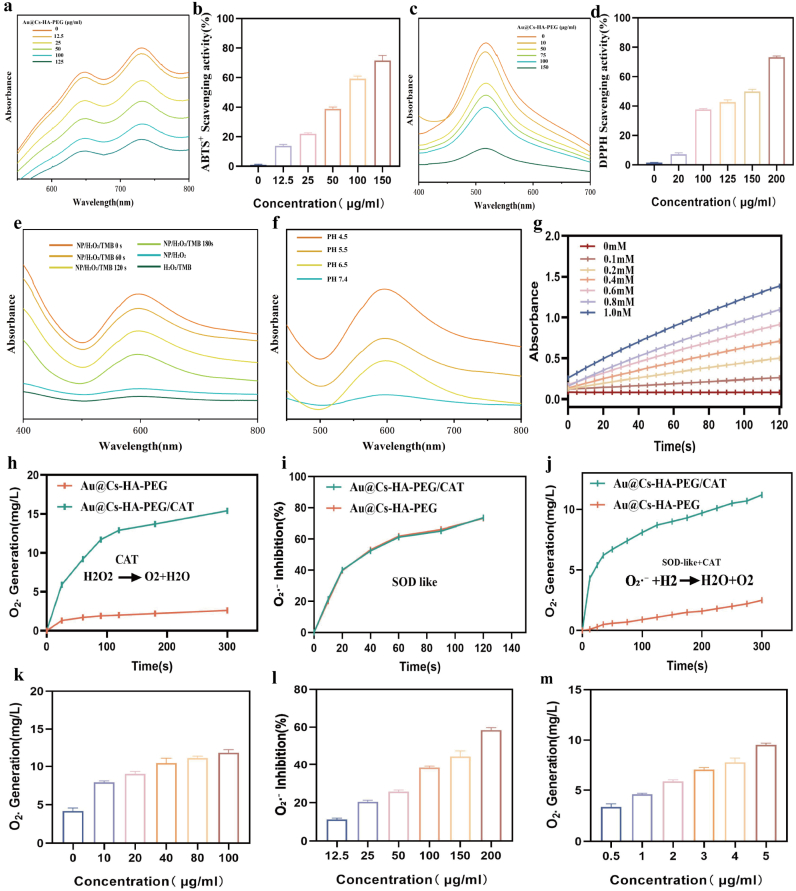
(a) Ultraviolet absorption spectra and quantitative analysis of ABTS at different nanometer concentrations (b). (c) Quantitative analysis of ultraviolet absorption spectra of DPPH at different nanometer concentrations (d). (e–g) The POD activity of Au@Cs -HA-PEG NPs was detected by changing the absorbance of the mixture containing Au@Cs -HA-PEG NPs, PH, H_2_O_2_ and TMB. (h, k) In vitro CAT activity of Au@Cs -HA-PEG/CAT. (i, l) In vitro SOD-like activity of Au@Cs -HA-PEG/CAT. (j, m) In vitro integrated cascade catalysis of Au@Cs -HA-PEG/CAT. The data are expressed as mean ± SD.

In the brains of Alzheimer's disease model mice, the lesion sites generate substantial amounts of reactive oxygen species (ROS), including O_2_·^-^, ·OH, and H_2_O_2_. However, although O_2_·^-^ constitutes the majority of ROS produced in AD, other free radicals continue to attack biomolecules. Therefore, the ability of Au@Cs–HA–PEG and Au@Cs–HA–PEG/CAT to scavenge other free radicals was evaluated. Their overall free radical scavenging activity was determined using 2, 2-diphenyl-1-picrylhydrazyl (DPPH) and 2,2′-azino-bis(3-ethylbenzothiazoline-6-sulfonic acid) (ABTS) radical assays. ABTS was oxidized in the presence of K_2_S_2_O_8_ to generate stable ABTS ^+^ radicals, which exhibited a characteristic UV–Vis absorption peak at 734 nm. Au@Cs–HA–PEG effectively reduced the UV–Vis absorbance, indicating its free radical scavenging capability (Fig. 3a and b). DPPH radicals showed a strong UV–Vis absorption peak at 517 nm, and the absorbance decreased with increasing concentrations of Au@Cs–HA–PEG, demonstrating it's radical scavenging ability (Fig. 3c and d). Furthermore, Au@Cs–HA–PEG effectively scavenged ·OH radicals as assessed in a reaction with 3,3′,5,5′-tetramethylbenzidine (TMB), showing a gradual decrease in UV–Vis absorbance (Fig. 3e). Considering that the CAT-like activity promotes the conversion of H_2_O_2_ to O_2_, thereby ameliorating the hypoxic environment in the Alzheimer's brain and enhancing therapeutic efficacy, we used an oxygen meter to measure oxygen levels and confirm the CAT-like activity of Au@Cs–HA–PEG. As shown in Fig. 3f, the Au@Cs–HA–PEG nanocomposite generated more O_2_ under acidic conditions. The production of dissolved oxygen was systematically evaluated over time under progressively acidic conditions. Additionally, we investigated the reaction kinetics, which were found to be dependent on both concentration and time (Fig. 3g). Similarly, The Au@Cs–HA–PEG nanoparticles exhibit superoxide dismutase (SOD)-like activity, enabling the conversion of toxic O_2_·^-^ into H_2_O_2_, indicating their potential therapeutic capability for Alzheimer's disease. The SOD-like activity of Au@Cs–HA–PEG NPs was investigated by monitoring their scavenging capacity for O_2_·^-^ generated in a xanthine/xanthine oxidase mixture. The results demonstrated that the nanoparticles possess SOD-like activity, which increased in a concentration-dependent manner (Fig. 3i–l). Subsequently, catalase (CAT) acts on hydrogen peroxide, converting it into harmless H_2_O and O_2_ (Fig. 3h–k). Finally, the impact of the entire cascade process was explored, and the results indicated that the CEPG/CAT combination was effective (Fig. 3j–m). Overall, the Au@Cs–HA–PEG/CAT cascade system exhibited strong scavenging effects on different types of free radicals. However, the addition of CAT had a limited impact on ROS clearance compared to Au@Cs–HA–PEG alone.

### 3.3 In vitro assessment of cellular uptake capacity and improvement of mitochondrial function

**Fig. 4:**
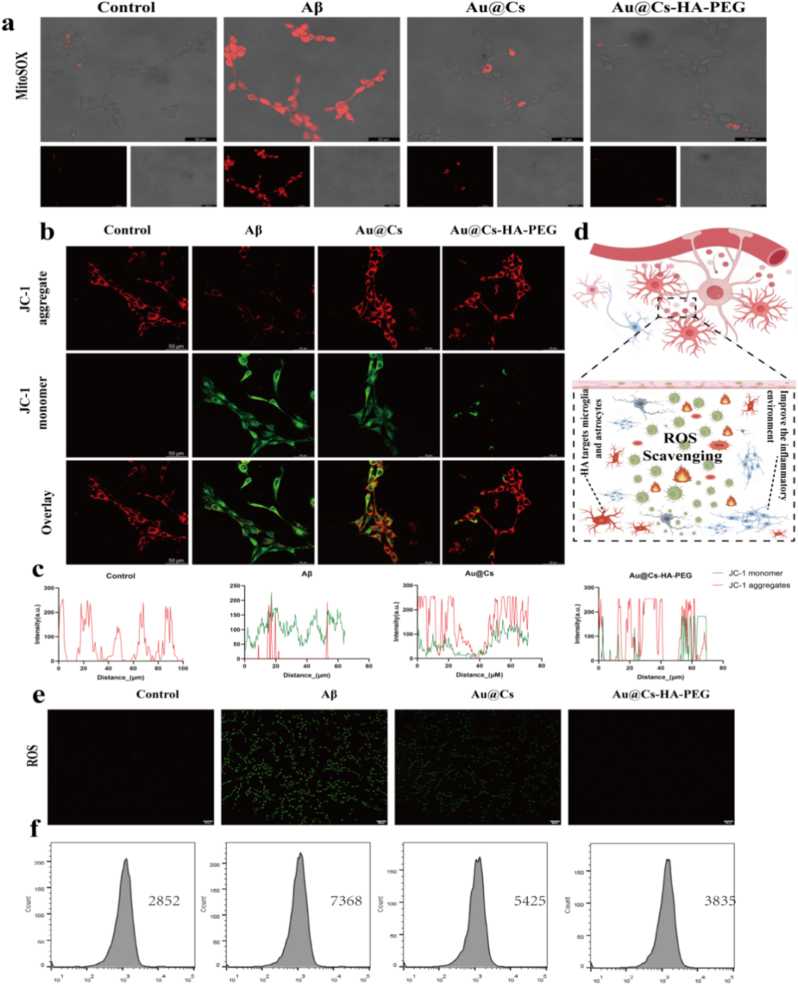
(a) Representative confocal fluorescence image of Mito-SOX staining, scale 50 μm. (b) Representative confocal fluorescence image of JC-1 staining, red, JC-1 aggregates; Green, JC-1 monomer. Scale, 50 μm. (c) Au@Cs-HA-PEG Schematic diagram of nanoparticles clearing reactive oxygen species within cells and reversing the state of neuronal cells. (d) The Pearson coefficients of the red JC-1 aggregates and the green JC-1 monomers. (e) Confocal microscopy showed the fluorescence intensity of the ROS probe DCFH-DA in HT22 cells. HT22 cells were pretreated with Au, Cs, Au@Cs and Au@Cs-HA-PEG (scale = 200 μm). (f) The fluorescence intensity of DCFH-DA was detected by flow cytometry. (For interpretation of the references to color in this figure legend, the reader is referred to the Web version of this article.)

Prior to conducting cellular experiments, we assessed the safety profile of Au@Cs-HA-PEG nanomedicines using the CCK-8 assay. As shown in Fig. S5, varying doses of Au@Cs-HA-PEG nanomedicines were administered to 5Y5Y, BV2, and HT22 cells, and their cytotoxic effects were evaluated. The results demonstrated that even at a concentration of 150 μg/mL, the cell viability remained above 95 %. Furthermore, we verified the capability of the nanomedicines to effectively scavenge intracellular reactive oxygen species (ROS). Mito-SOX staining, which specifically detects mitochondrial ROS, revealed that Au@Cs-HA-PEG treatment most effectively suppressed mitochondrial ROS production. As shown in Fig. 4a, oxidative stress can induce mitochondrial membrane potential depolarization and phospholipid oxidation-dependent membrane rupture. JC-1 staining demonstrated that Aβ stimulation reduced the red fluorescent JC-1 aggregates, indicative of depolarized mitochondria, while Au@Cs-HA-PEG treatment markedly restored red fluorescence and attenuated green monomeric JC-1 fluorescence (Fig. 4b and c). Notably, Au@Cs-HA-PEG was more effective than Au@Cs in rescuing Aβ-induced mitochondrial membrane potential collapse (Fig. 4d). Using the fluorescent probe DCFH-DA, we quantified intracellular ROS levels. Both inverted microscopy and flow cytometry analyses revealed a significant increase in ROS in HT22 cells upon Aβ exposure. However, co-treatment with Au@Cs or Au@Cs-HA-PEG markedly reduced ROS levels, as illustrated in Fig. 4e and f.

### 3.4 In vitro evaluation of the clearance of intracellular inflammatory factors and anti-apoptosis

**Fig. 5:**
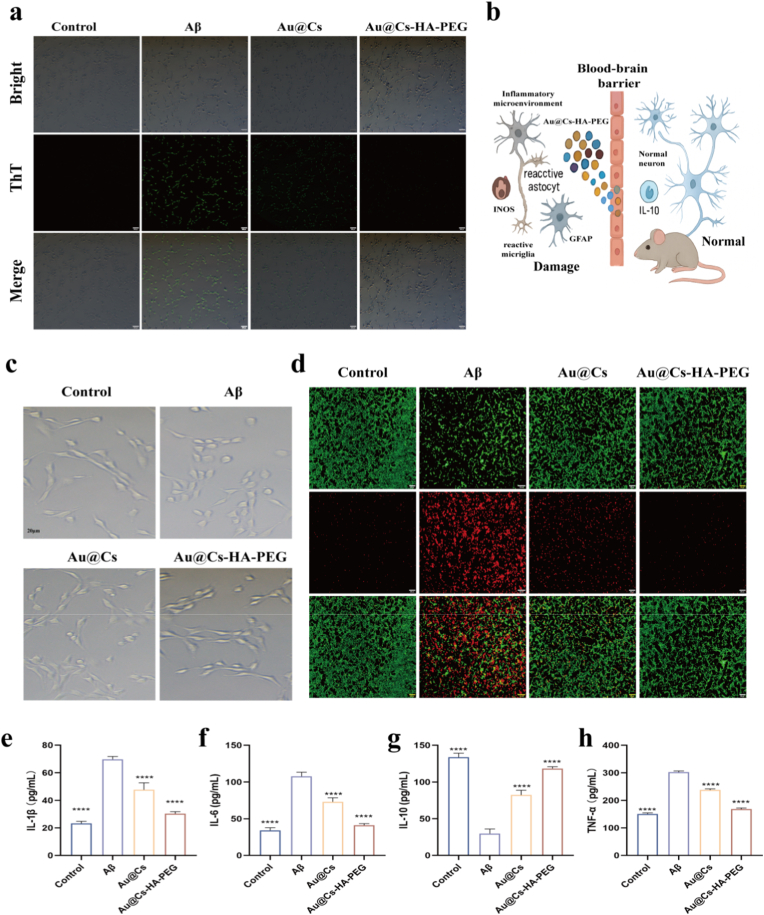
(a) Intracellular ThT fluorescence intensity in confocal FL images (scale: 200 μm). (b) Au@Cs-HA-PEG nanoparticles cross the blood-brain barrier in the mouse brain to inhibit the outbreak of inflammatory factors in the brain. (c) The state of HT22 cells after treatment with different nanomedicines. (d) Images of HT22 cells treated with live/dead staining (scale: 100 μm). Changes in the contents of (e) IL-1β (f), IL-6 (g), IL-10, and (h) TNF-α in HT22 cells after treatment with different nanomedicines. The data are expressed as mean ± SD. ∗∗∗p < 0.001, ∗∗p < 0.01.

In the early stages, excessive extracellular deposition of amyloid protein leads to microglial over activation and substantial intracellular generation of reactive oxygen species (ROS). Mitochondria, which are primarily responsible for converting energy from nutrients into adenosine triphosphate (ATP) via cellular respiration, serve as the powerhouse of the cell. Excessive intracellular ROS production disrupts nutrient uptake, ultimately leading to cellular apoptosis. We investigated the role of Au@Cs-HA-PEG in degrading Aβ fibrils. HT22 cells were co-incubated with Aβ fibrils, and changes in Thioflavin T (ThT) fluorescence intensity were assessed using laser confocal microscopy. As shown in Fig. 5a and Fig. S10, abundant green ThT fluorescence was observed in HT22 cells cultured with Aβ fibrils, indicating fibril formation. In contrast, fluorescence was nearly absent in the Au@Cs-HA-PEG-treated group, demonstrating disruption of the β-sheet structure and confirming the ability of Au@Cs-HA-PEG to inhibit Aβ aggregation at the cellular level Fig. 5b illustrates that Au@Cs-HA-PEG nanomedicines can effectively clear inflammatory factors—IL-1β, IL-6, and TNF-α—in the brain after crossing the blood-brain barrier. To evaluate the therapeutic effect of Au@Cs-HA-PEG on cellular morphology, we captured images of HT22 cells under various treatments using confocal laser scanning microscopy (CLSM). Following treatment with Au@Cs and Au@Cs-HA-PEG, Aβ-stimulated HT22 cells, which had adopted a deformed spherical shape, tended to revert toward a spindle-like morphology (Fig. 5c). Notably, recovery was most pronounced in the Au@Cs-HA-PEG treatment group. The inhibitory effect of Au@Cs-HA-PEG on Aβ-induced HT22 cell apoptosis was further corroborated by AM/PI double staining. As shown in Fig. 5d, incubation with Aβ resulted in numerous red fluorescence signals, indicative of apoptotic cells. In contrast, the Au@Cs-HA-PEG group showed significantly reduced red fluorescence, suggesting effective protection against amyloid-related cytotoxicity. Additionally, we assessed the anti-inflammatory properties of Au@Cs-HA-PEG using supernatants from cultured BV2 microglia. As shown in Fig. 5e–h, co-incubation with Aβ significantly stimulated BV2 cells to secrete pro-inflammatory cytokines (TNF-α, IL-1β, and IL-6) and suppressed production of the anti-inflammatory cytokine IL-10. However, when BV2 cells were co-cultured with Au@Cs-HA-PEG for 12 h, levels of TNF-α, IL-1β, and IL-6 decreased, while IL-10 levels increased markedly. In summary, Au@Cs-HA-PEG effectively scavenges intracellular ROS, inhibits Aβ aggregation, suppresses apoptosis, and protects neuronal cells.

**Fig. 6:**
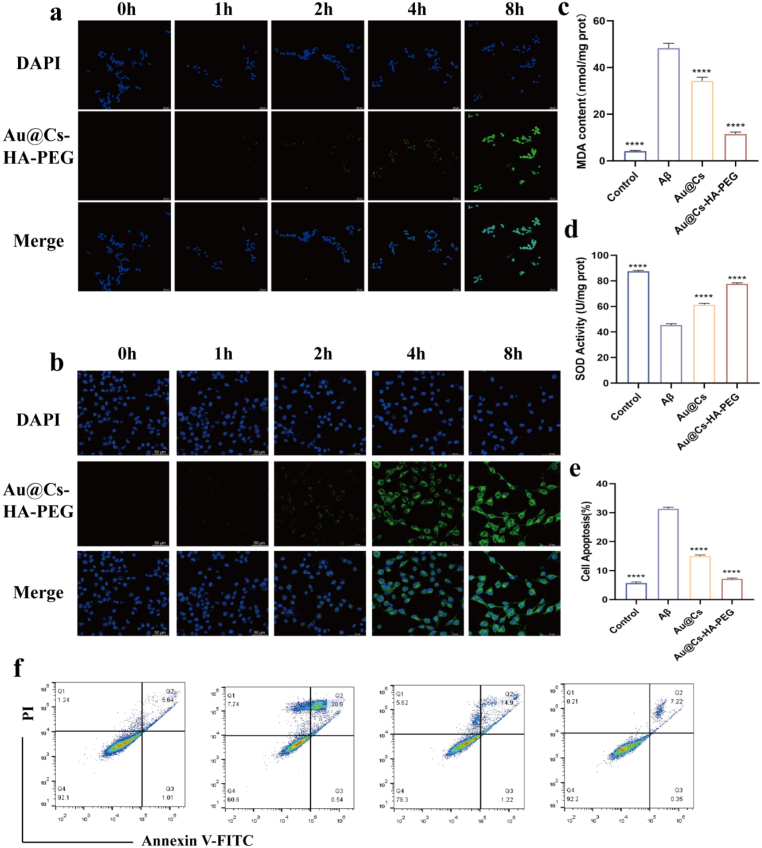
(a) Uptake of FITC-Au@Cs-HA-PEGNPs in BV2 cells at different time points (scale: 50 μm). (b) Uptake of FITC-Au@Cs-HA-PEGNPs in HT22 cells at different time points (scale: 50 μm). The contents of (c) MDA and (d) SOD in cells after treatment with Au@Cs and Au@Cs-HA-PEG. Data are expressed as mean ± SEM(n = 3). (e) Quantitative analysis of apoptosis rate. (f) Apoptosis of HT22 cells was detected by Annexin V/PI double staining. The data are expressed as mean ± SD. ∗P < 0.05, ∗∗P < 0.01, ∗∗∗P < 0.001, and ∗∗∗∗P < 0.0001.

To evaluate the cellular uptake efficiency and intracellular retention time of Au@Cs and Au@Cs-HA-PEG in target cells, an in vitro blood–brain barrier (BBB) model was established via co-culture. The integrity of the BBB model was confirmed by measuring transendothelial electrical resistance. In Fig. S11, fluorescein isothiocyanate-labeled Au@Cs-HA-PEG nanomaterials were incubated with hippocampal neuron (HT22) and microglial (BV2) cells for different durations. The results confirmed the successful and time-dependent internalization of Au@Cs-HA-PEG into both HT22 (Fig. 6b) and BV2 (Fig. 6a) cells, indicating efficient uptake by the target cells, and conduct quantitative analysis on it (Figs. S6 and 7). Additionally, we confirmed that the nanomaterials could target overactivated microglia and astrocytes upon crossing the BBB, leveraging the abundant surface expression of CD44, which specifically binds hyaluronic acid. We also measured the levels of malondialdehyde (MDA), a marker of lipid peroxidation, and found that Au@Cs-HA-PEG significantly inhibited Aβ-induced MDA production (Fig. 6c). Similarly, the nanomedicine counteracted the Aβ-induced reduction in superoxide dismutase (SOD) activity (Fig. 6d). As shown in Fig. 6e and f, apoptosis rates increased from 5.64 % in the control group to 30.9 % after Aβ treatment. In addition, we detected the anti-apoptotic ability of Au@Cs-HA-PEG nanoparticles by verifying the expression levels of Bax (Fig. S9) and Bcl-2 (Fig. S8) proteins in cells through cellular immunofluorescence assay. Treatment with Au@Cs and Au@Cs-HA-PEG reduced apoptosis rates to 14.9 % and 7.22 %, respectively.

### 3.5 Evaluation of brain targeting and in vivo safety of Au@Cs-HA-PEG

**Fig. 7:**
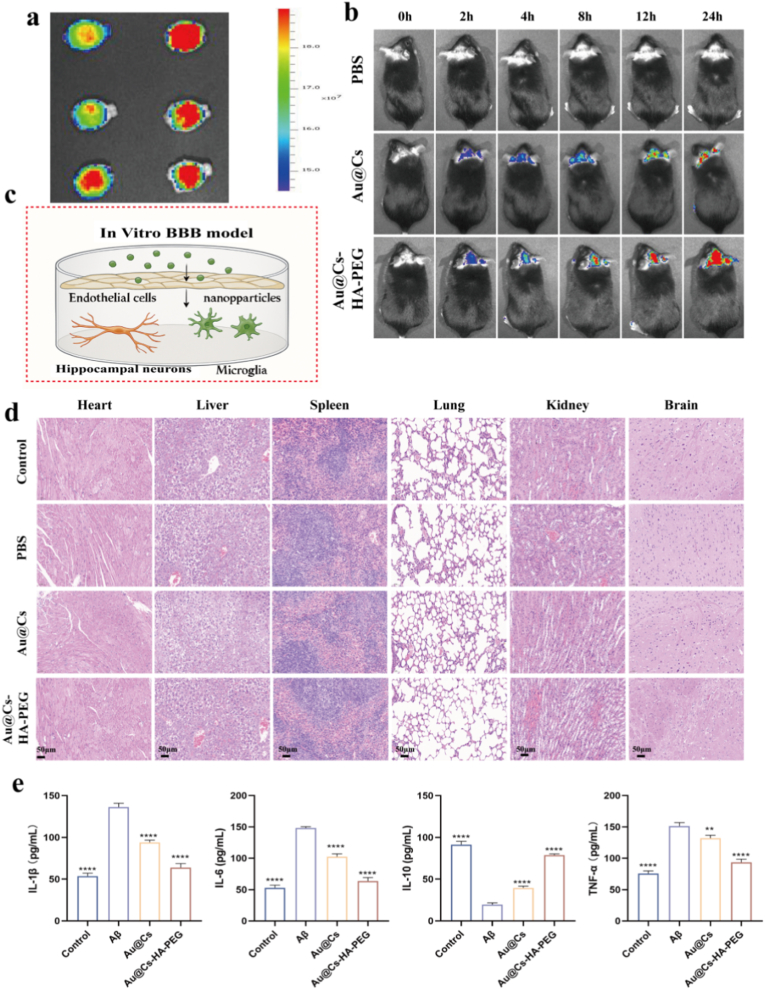
(a) In vitro fluorescence imaging of the brain 24 h after administration of pbs, Au@Cs, Au@Cs-HA-PEG. (b) Intracranial fluorescence images of C57 mice after administration of different formulations (pbs, Au@Cs, Au@Cs-HA-PEG) for 0, 2, 4, 8, 12, and 24 h. (c) Schematic diagram of the in vitro BBB model. (d) Hematoxylin and eosin (H&E) staining of major organs after injection with different nanoparticles (pbs, Au@Cs, Au@Cs-HA-PEG) (scale = 50 μm). (e) Quantitative analysis of TNF-α, IL-1β, IL-10 and IL-6 in the brain tissues of WT and AD mice after different drug treatments. The data are expressed as mean ± SD. ∗∗∗p < 0.001, ∗∗p < 0.01.

Prior to in vivo experiments, we assessed the biocompatibility of Au@Cs-HA-PEG using hemolysis assays. The results indicated that after 4 h of co-incubation, the red blood cell membranes remained intact, with a hemolysis rate below 5 % for all samples (Fig. S12), demonstrating favorable hemocompatibility of the nanoparticles. To evaluate the brain accumulation and biodistribution of Au@Cs-HA-PEG, FITC-labeled nanomedicine was administered via tail vein injection in mice (Fig. 7b). In vivo imaging at various time points revealed a time-dependent increase in brain distribution of Au@Cs-HA-PEG, peaking at 24 h post-injection. During the same period, only minimal amounts of unmodified Au@Cs crossed the blood-brain barrier. Ex vivo fluorescence imaging of isolated major organs confirmed that the fluorescence patterns in brain tissue were consistent with the in vivo observations. As shown in Fig. 7a, substantial accumulation of Au@Cs-HA-PEG and minimal accumulation of Au@Cs were detected in the brain 48 h after administration. Furthermore, in vivo imaging of the heart, liver, spleen, and kidneys showed higher fluorescence intensity in the liver and kidneys of mice treated with Au@Cs compared to those treated with Au@Cs-HA-PEG (Fig. S16). To further evaluate the biocompatibility of Au@Cs-HA-PEG, histological analysis was performed on major organs (heart, liver, spleen, and kidney) of C57 mice injected with the nanocomposite. No obvious pathological abnormalities were observed in the Au@Cs-HA-PEG group compared to the PBS control group (Fig. 7d). Fig. 7c schematically illustrates how Au@Cs-HA-PEG nanomedicines cross the blood-brain barrier in mice and are taken up by hippocampal neurons and microglia. Given that neuroinflammation is closely linked to Alzheimer's disease (AD) progression, we used ELISA to measure levels of key pro-inflammatory cytokines (TNF-α, IL-1β, IL-6) and the anti-inflammatory cytokine IL-10 in the brains of AD mice. As shown in Fig. 7e, TNF-α, IL-1β, and IL-6 were significantly elevated, while IL-10 was markedly reduced in AD mice. Treatment with Au@Cs-HA-PEG significantly reduced the levels of pro-inflammatory factors and increased IL-10, confirming its anti-inflammatory efficacy. These results suggest that reduced Aβ accumulation contributes to decreased ROS and inflammation. In conclusion, Au@Cs-HA-PEG nanocarriers exhibit strong antioxidant, anti-inflammatory, and neuroprotective properties, highlighting their therapeutic potential for Alzheimer's disease.

### 3.6 Au@Cs-HA-PEG improves cognitive impairment and learning ability in mice

**Fig. 8:**
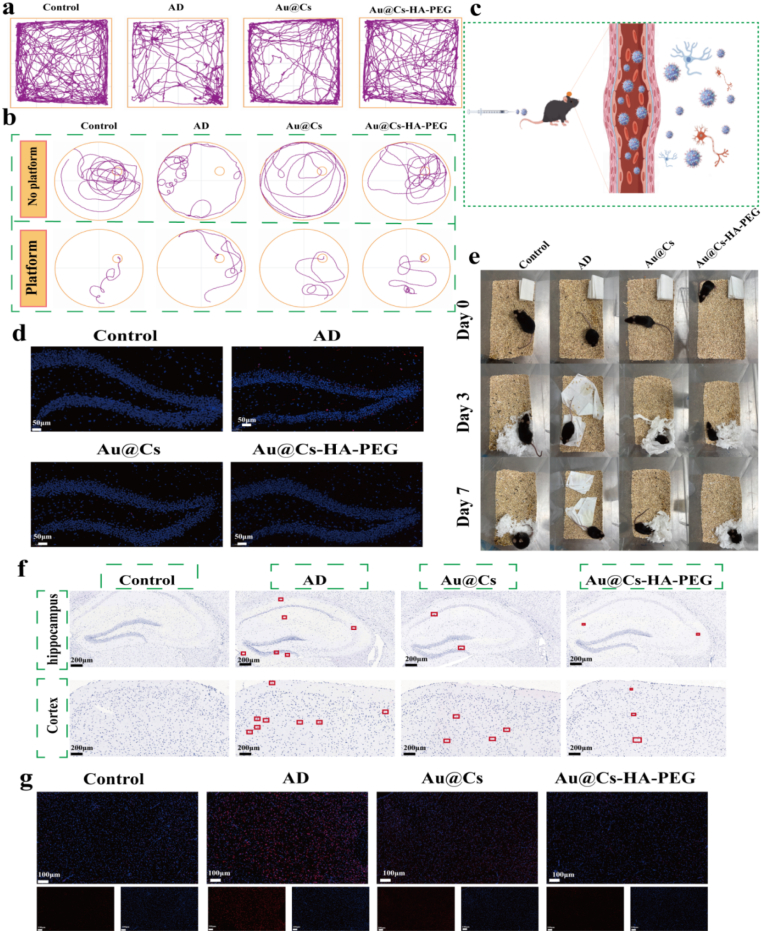
(a) Swimming path diagram of mouse MWM experiment. (b) Path diagram of the mouse open field experiment. (c) Au@Cs-HA-PEG Schematic diagram of crossing the blood-brain barrier and targeting neurons and microglia. (d) Immunofluorescence images of Aβ in the brains of mice after treatment with different nanomaterials. (e) Representative images of nesting behavior on day 0, day 2 and day 4. (f) Analysis of Congo red staining of Aβ plaques in brain tissue: WT mice and AD mice were treated with pbs, Au@Cs and Au@Cs-HA-PEG, respectively. (g) Immunofluorescence analysis of ROS in the brains of mice after treatment with different nanomedicines. (For interpretation of the references to color in this figure legend, the reader is referred to the Web version of this article.)

**Fig. 9:**
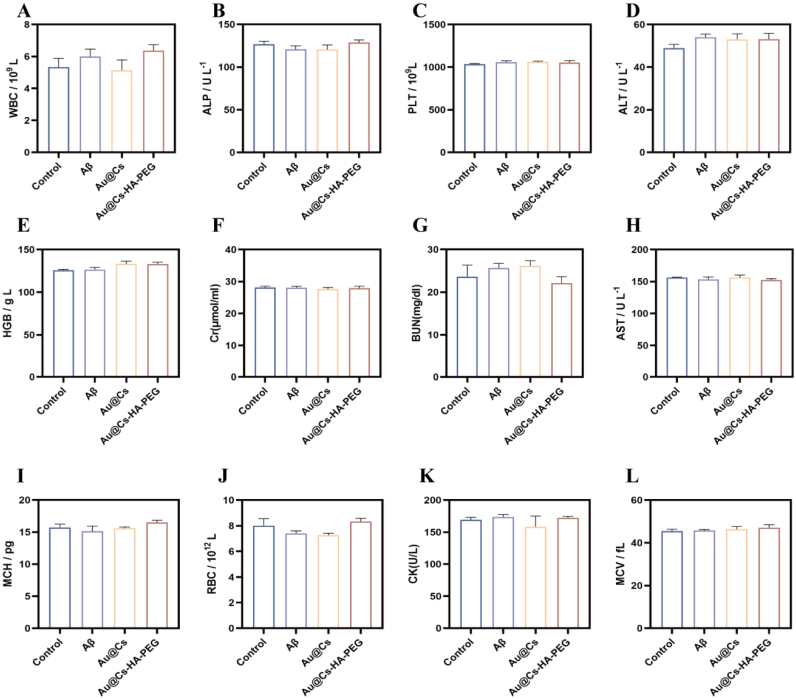
Blood biochemistry analysis of WT mice and AD mice injected with different treatments via tail vein (A) WBC: leukocyte (B) ALP: alkaline phosphatase (C) PLT: platelet (D)ALT: alanine aminotransferase (E) HGB: hemoglobin (F): Creatinine (G) BUN: blood urea nitrogen (H) AST: aspartate aminotransferase (I) MCH: hemoglobin content (J) RBC: erythrocyte (K): Creatine kinase (L) MCV: mean corpuscular volume. The data are expressed as mean ± SD.

Behavioral experiments were performed using 8-month-old APP/PS1 transgenic Alzheimer's disease (AD) model mice, with age-matched wild-type C57 mice serving as the control group. After one month of continuous treatment, during which body weight was monitored, no significant fluctuations were observed (Fig. S13). We subsequently conducted behavioral assessments. The real-time movement trajectories over a 5-min period are shown in Fig. 8a. Compared to WT mice, AD mice exhibited simplified exploratory behavior, primarily lingering near the edges of the arena with reduced exploratory drive. Following treatment with Au@Cs or Au@Cs-HA-PEG nanomedicines, the movement patterns became more complex and exploratory. As shown in Figure S14E and Figure S14F, both the duration and distance of activity in the central zone increased after nanomedicine treatment. In the Morris water maze (MWM) test, mice underwent 5 days of training. On the test day, two trials were conducted: one with the platform removed and another with the platform repositioned. Representative swimming paths are illustrated in Fig. 8b. Even after training, AD mice displayed significant memory impairment, characterized by difficulty locating the platform and disorganized swimming. In contrast, mice treated with Au@Cs or Au@Cs-HA-PEG exhibited more directed swimming and improved spatial learning. As shown in Figure S14B and Figure S14C, AD mice demonstrated the longest escape latency and the shortest cumulative time and distance in the target quadrant, indicating impaired spatial memory. Both nanomedicine treatments ameliorated these deficits, with the Au@Cs-HA-PEG group showing the most pronounced improvement. The average swimming speed of the mice was also improved in the Au@Cs-HA-PEG group (Fig. S14D). The number of platform crossings—a key indicator of spatial memory—was significantly higher in treated groups, particularly with Au@Cs-HA-PEG (Fig. S14A), suggesting a restoration of cognitive function. Nesting behavior, assessed as shown in Fig. 8e, was also improved in both treatment groups compared to the AD group, with the Au@Cs-HA-PEG group showing better performance on days 2 and 4. Fig. 8c illustrates the proposed mechanism: following tail vein injection, Au@Cs-HA-PEG crosses the blood-brain barrier and targets inflamed microglia and astrocytes. After behavioral testing, mice were euthanized for brain tissue analysis. Immunofluorescence staining revealed extensive Aβ deposition in the hippocampus of AD mice, evident as abundant fluorescent plaques, in stark contrast to WT mice (Fig. 8d). Nanomedicine treatment markedly reduced Aβ burden. Congo red staining further confirmed a significant reduction in Aβ deposition across treatment groups (Fig. 8f). In addition, the Caspase-3 protein staining of mouse brain slices was used to observe the evaluation of the anti-apoptotic ability of Au@Cs-HA-PEG nanoparticles in the mouse brain (Fig. S15). We also detected ROS levels in brain sections (Fig. 8g). As expected, AD mice exhibited intense red fluorescence, indicating high ROS levels, which were substantially higher than in WT mice. Au@Cs-HA-PEG treatment significantly attenuated oxidative stress, confirming that the nanomedicine confers neuroprotection by mitigating apoptosis and reducing oxidative damage. Subsequently, we sacrificed the mice for biosafety testing. The hematological parameters, including WBC, RBC, ALP, ALT, PLT, HGB, AST, BUN, MCH, MCV, Cr, and ck, were all within the normal range Fig. 9a–l, supporting the biosafety of the material.

## 4 Conclusions

In this study, we successfully developed a multifunctional composite nanodrug—Au@Cs-HA-PEG—for the treatment of Alzheimer's disease (AD), marking a new era of precision therapy in AD-targeted treatment strategies. This nanosystem innovatively leverages the specific interaction between hyaluronic acid (HA) and CD44 receptors, which are highly expressed on microglia and astrocytes under neuroinflammatory conditions, enabling precise and active targeting of affected brain regions. The HA modification not only facilitates efficient traversal of the nanoparticles across the blood-brain barrier but also significantly enhances drug bioavailability, highlighting its superiority as a high-performance brain-targeted delivery platform. The formulation exhibits outstanding stability and favorable biocompatibility, while the outer HA layer effectively prolongs systemic circulation time, laying a solid foundation for sustained therapeutic efficacy. Through comprehensive in vitro and in vivo experiments, Au@Cs-HA-PEG has demonstrated multiple disease-modifying capabilities: effectively inhibiting Aβ production, promoting the clearance of reactive oxygen species (ROS), and reversing neuronal dysfunction, thereby enabling multi-dimensional intervention of key AD pathological processes. These findings indicate that Au@Cs-HA-PEG represents a promising candidate for clinical AD therapy.

## CRediT authorship contribution statement

**Chaonan Jing:** Formal analysis, Data curation, Conceptualization. **Junjie Li:** Formal analysis, Data curation, Conceptualization. **Dehong Yu:** Formal analysis, Data curation. **Minghao Chao:** Formal analysis, Data curation. **Hanrong Yan:** Formal analysis, Data curation, Conceptualization. **Kezhen Ge:** Formal analysis, Data curation. **Guangyu Ma:** Formal analysis, Data curation. **Jiangbo Wang:** Formal analysis, Data curation. **Fenglei Gao:** Visualization, Validation, Supervision. **Guanqun Zhang:** Formal analysis, Data curation.

## Ethics declarations

All animal experiments were approved by the Animal Protection and Ethics Committee of Xuzhou Medical University. The ethical code of animal study is 202411T024.

## Declaration of competing interest

The authors declare that they have no known competing financial interests or personal relationships that could have appeared to influence the work reported in this paper.

## Data Availability

Data will be made available on request.
