# Supplementary material for: Heterotrimetallic Au@Cu2Se nanozymes target inflamed neurons via suppression of oxidative stress and apoptosis to alleviate Alzheimer's disease
Source: Mater Today Bio. 2025 Dec 7;36:102646. doi: 10.1016/j.mtbio.2025.102646 (PMC12741421; doi:10.1016/j.mtbio.2025.102646)
Supplement: Multimedia component 1 [file mmc1.docx]

**Supporting Information**

**Heterotrimetallic Au@Cu₂Se Nanozymes Target Inflamed Neurons via Suppression of Oxidative Stress and Apoptosis to Alleviate Alzheimer’s Disease**

Chaonan Jing^a,b,c,1^, Junjie Li^c,1^, Dehong Yu^d,1^, Minghao Chao^e,1^, Hanrong Yan^c^, Kezhen Ge^c^, Guangyu Ma^c^, Jiangbo Wang^a,b*^, Fenglei Gao^c,*^, and Guanqun Zhang^a,b,*^

a. Department of Neurology, Xuzhou Clinical School of Xuzhou Medical University, Xuzhou, Jiangsu 221002, China.

b. Department of Neurology, Xuzhou Central Hospital, Xuzhou, Jiangsu 221002, China.

c. Jiangsu Key Laboratory of New Drug Research and Clinical Pharmacy, Xuzhou Medical University, Xuzhou, Jiangsu 221004, China.

d. Department of Oncology, The Affiliated Pizhou Hospital of Xuzhou Medical University, Pizhou, Jiangsu, 221399, China.

e. Department of Orthopaedic Surgery, Lishui Central Hospital and Fifth Affiliated Hospital of Wenzhou Medical University, Lishui, 323000, Zhejiang, China

^1^These authors contributed equally to this research work.

*Corresponding Author. Email: [zgq18@sina.com (G. Zhang)](mailto:zgq18@sina.com%20(G.%20Zhang)), flgao@xzhmu.edu.cn (F. Gao), [wangjiangbo2011@126.com](mailto:wangjiangbo2011@126.com) (J. Wang).

**Contents**

**Preparation of Aβ solution. S4**

**Live/dead cell assay. S4**

**Au@Cs-HA-PEG intake experiment S4**

**CCK-8 experiment. S4**

**Au@Cs-HA-PEG targeting evaluation. S5**

**Analysis of reactive oxygen species. S5**

**Hemolysis test. S5**

**Behavioral experiment. S5**

**Nesting experiment. S6**

**ThT fluorescence determination. S6**

**Enzyme-linked immunosorbent assay (ELISA). S6**

**Assessment Peroxidase (POD) Activity S7**

**Assessment Peroxidase (POD) Activity. S7**

**Statistical analysis. S7**

**SEM images of Au@Cs NPs (Figure S1) S8**

**TEM image of Au@Cs-HA-PEG NPs (Figure S2) S8**

**Stability of Au@Cs and Au@Cs-HA-PEG (Figure S3) S9**

**FTIR (Figure S4) S10**

**CCK-8 (Figure S5) S11**

**Uptake rate of BV2 and HT22 (Figure S6, 7) S11**

**Fluorescence images of Bcl-2 (Figure S8) S12**

**Fluorescence images of Bax (Figure S9) S12**

**THT fluorescence (Figure S10) S13**

**TEER values (Figure S11) S13**

**Hemolysis rate (Figure S12) S14**

**Body weight changes of mice (Figure S13) S14**

**MWM and open field experiment date (Figure S14) S15**

**Immunofluorescence analysis of Caspase-3 (Figure S15) S16**

**In vitro fluorescence images (Figure S16) S16**

**Reference S17**

**Preparation of Aβ solution.** A β1−42 monomer (1 mg) was dispersed in hexafluoroisopropanol (HFIP) (1mL); then seal the container and shake it at 4 ℃ for 2 hours. When used again, remove HFIP from the original solution, evaporate it, and then mix it with PBS buffer to form an Aβ solution. After 1 minute of ultrasonic treatment, continuous oscillation at 37 ℃ for 7 days was carried out to form aβ fibers.

**Live/dead cell assay.** The therapeutic effect of Au@Cs-HA-PEG on HT22 cells was assessed using the Calcineurin-AM/PI Live/Dead Cell Assay Kit. Specifically, HT22 cells were inoculated at 3 × 10^5^ per well into a 6-well plate and incubated at 37 °C for 24 h until confluency reached approximately 80 %. Subsequently, the cells were treated in different ways for 12 h. The cells were stained with calcein-AM/PI working solution for 20 min at 37 °C in the dark and then observed under an inverted fluorescence microscope (Olympus IX73, Japan).

**Au@Cs-HA-PEG intake experiment.** 80,000 HT22 cells were inoculated in confocal dishes. Fitc-labeled Au@Cs-HA-PEG solution (125 μg/mL, 100 μL) was added at 0, 2, 4, and 6 hours before shooting, and DAPI staining was performed for 15 minutes. Subsequently, examine the plates under a confocal laser scanning microscope (CLSM, Leica STELLARIS 5, Germany). Confocal microscopy Settings are as follows: (1) DAPI channel: Excitation wave length 405 nm, exposure time 200 ms. (2) FITC channel: Excitation wavelength 488nm, exposure time 500ms. (3) Objective lens magnification: 40x objective lens.

**CCK-8 experiment.** HT22, 5Y5Y, and BV2 cells were inoculated in 96-well plates, with 10,000 cells per well. They were treated with different concentrations of Au@Cs-HA-PEG solution for 3/6/12/24 hours and stained with CCK-8 solution (10% CCK-8 dye + 90% DMEM medium). The absorbance at 540nm was measured using a microplate instrument to evaluate the cytotoxicity of Au@Cs-HA-PEG against HT22, 5Y5Y, and BV2 cells[1].

**Au@Cs-HA-PEG targeting evaluation.** Au@Cs-HA-PEG (5 mM, FITC-labeled) injected via tail vein in mice. The small animal imaging system was used to monitor its targeting effect on the brain tissue of AD mice and in vivo metabolism at 0, 2, 4, 8, 12 and 24 hours after injection[2].

**Analysis of reactive oxygen species.** HT22 cells were treated in serum-free medium with a β monomer and incubated at 37 ° C for 24 hours to induce ROS generation. The fluorescence intensity of the DCFH-DA probe (oxidation-sensitive fluorescent probe) represents the relative level of peroxides in cells. After treatment with pbs, Au@Cs, and Au@Cs-HA-PEG, the fluorescence intensity of DCFH-DA was observed under confocal microscopy, and its fluorescence intensity was quantitatively analyzed by flow cytometry (excitation wavelength 488 nm, emission wavelength 530 nm).

**Hemolysis test.** Centrifuge the fresh blood of C57 mice and wash repeatedly with PBS until the liquid becomes clear. About 2% of the red blood cell suspension was prepared with PBS and mixed with different concentrations of Au@Cs-HA-PEG nanocomposites (PBS, 50-400μg/mL). Deionized water was added as the positive control, and the pbs group was used as the negative control. The mixture was well mixed, and the solution was stored at 37 °C for 3 hours to observe the color change. After 6 hours, the supernatant was added to a 96-well plate and detected at a wavelength of 414 nm using an enzyme-linked immunosorbent assay (ELISA) reader. This process was repeated three times[3].

**Behavioral experiment.** The treatment subjects were 8-month-old APP/PS1 and C57 mice. Mice were treated with Au@Cs-HA-PEG nanomedicine by tail vein injection for 4 weeks. After the last administration, training and testing were conducted in the Morris Water Maze (MWM). The circular pool is divided into four quadrants, in which a hidden platform is placed. During the first five days, each mouse was trained four times in four quadrants. The mice were allowed to swim freely in water for 60 seconds or stop after finding a platform, and the incubation period required to climb the platform was recorded. After the last day of training, the platform was removed and the time the mice spent in the target quadrant for 60 seconds was recorded[4].

**Nesting experiment.** Before the experiment began, the mice were divided into several groups and raised for one week respectively. Place two tissues in the cage and record the nesting situation with a camera on the 0th, 2nd and 4th days. The specific quality of the nest is based on the following standards: 1 to 4 1. There is no obvious paper biting or tearing, and no identifiable nest address; 2. There are no obvious bite marks/tear marks on the paper and no identifiable nest sites. 3. Some bite mark/tear mark paper with identifiable nest sites; 4. The sharpest paper/torn paper, with identifiable nest positions.

**ThT fluorescence determination.** Cells were co-cultured with a β for 24 hours. After treatment with a combination of Au@Cs and Au@Cs-HA-PEG nanomedicines, they were transferred to a medium containing the nanosystem and incubated for 48 hours. They were fixed with paraformaldehyde and co-stained with ThT and DAPI solutions. Next, thoroughly rinse the sample with PBS and observe the results under a fluorescence microscope. Record the fluorescence intensity and calculate the polymerization inhibition rate.[5]

**Enzyme-linked immunosorbent assay (ELISA).** Mouse brain tissue in a solution containing 50 mM Tris-HCl buffer (pH 7.4), 150 mM NaCl, 0.5% NP-40, 0.5% sodium deoxycholate, and 0.25% sodium dodecyl sulfate Homogenize 5 mM EDTA and protease inhibitor (100,000 g, 1 h, 4 °C) in cold lysis buffer. Take the supernatant and analyze the contents of IL-1β, IL-6, IL-10 and TNF-α in the brain according to the instructions of the relevant kit manufacturers.[6]

**Assessment Peroxidase (POD) Activity.** Prepare an 18 mM TMB solution and add it to three 5 mL centrifuge tubes. To Tube 1, add 1 mL of pH 5.5 PBS, 1 mL of 30% hydrogen peroxide, 100 μL of 1 mg/mL Au@Cs-HA-PEG solution, and 10 μL of the TMB solution. For Tube 2, add 1.01 mL of pH 5.5 PBS, 1 mL of 30% hydrogen peroxide, and 100 μL of 1 mg/mL Au@Cs-HA-PEG solution. To Tube 3, add 1.1 mL of pH 5.5 PBS, 1 mL of 30% hydrogen peroxide, and 10 μL of TMB solution. Using a UV spectrophotometer, measure the absorbance of the mixture in Tube 1 at 0, 60, and 120 seconds. The characteristic absorption peak of TMB near 650 nm should gradually decrease. Subsequently, measure the absorbance of Tubes 2 and 3.

**Evaluation Catalase (CAT) Activity.** Dilute 1 mL of 30% hydrogen peroxide with 6.5 mL of pH 5.5 PBS and add 100 μL of 1 mg/mL Au@Cs-HA-PEG solution, mixing thoroughly. Measure the absorbance of the solution at 0 and 120 seconds using a UV spectrophotometer, where a decrease in the hydrogen peroxide absorption peak at 240 nm can be observed.[7]

**Statistical analysis.** All quantitative results were obtained from at least 3 samples. Graphs were created and statistical data were analyzed using Origin 2022. Comparisons between the two groups were made by unpaired t-tests. Statistical significance was set at *p < 0.05, **p < 0.01, ***p < 0.001.


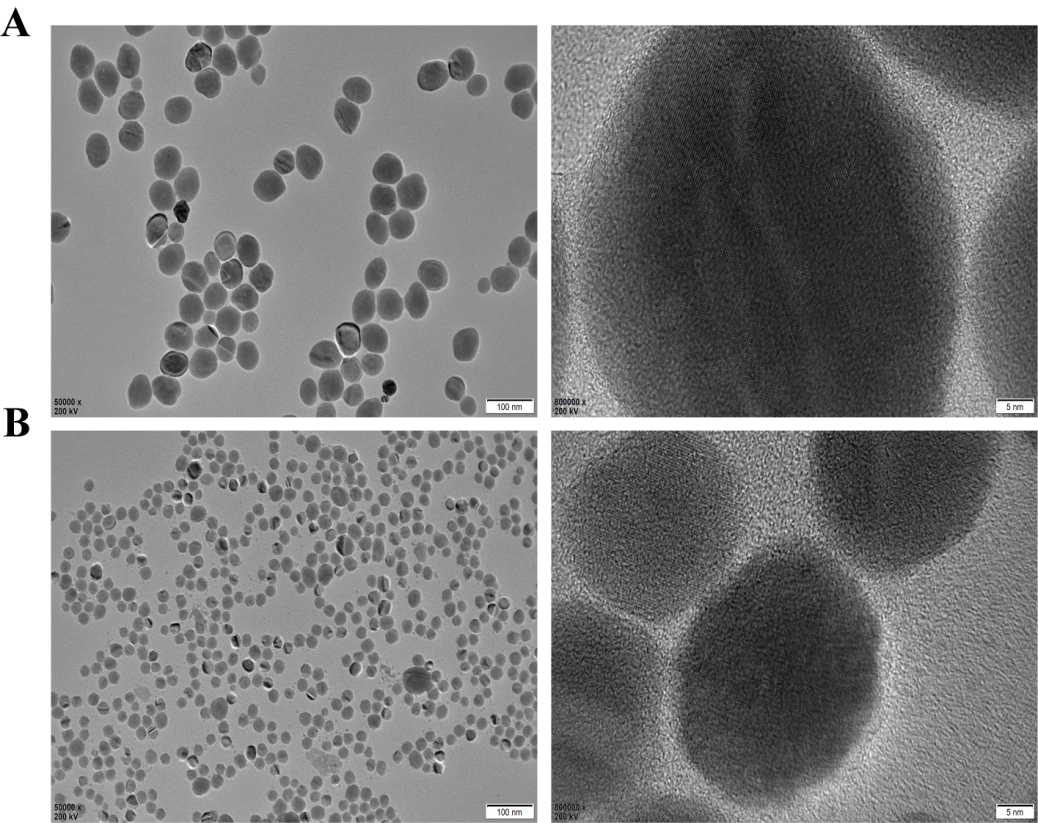
**Figure S1.** SEM images of SRT NPs (scale bar: 100 nm).

**
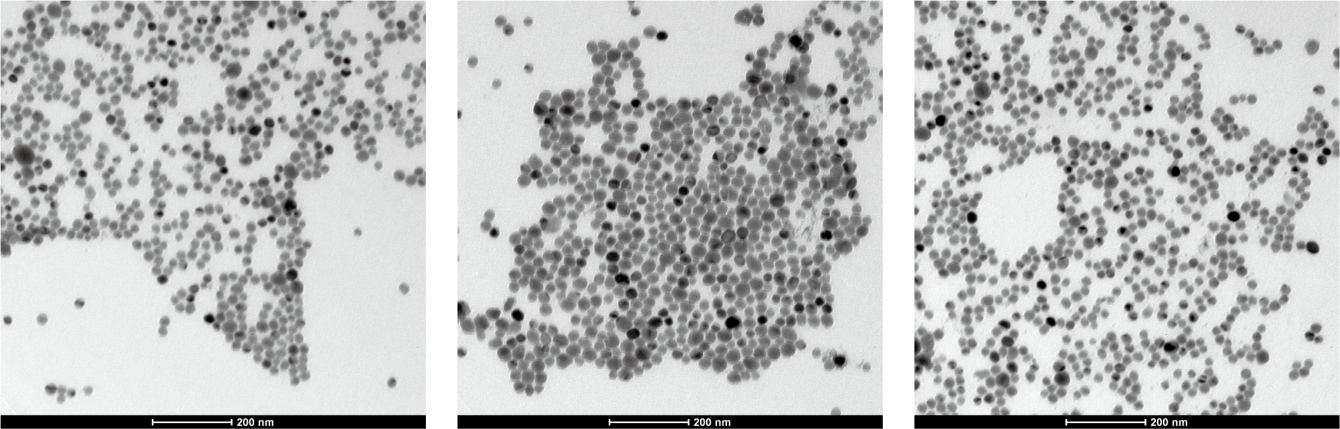
**

**Figure S2.** TEM image of Au@Cs-HA-PEG after 7 days (scale bar: 200 nm).

**
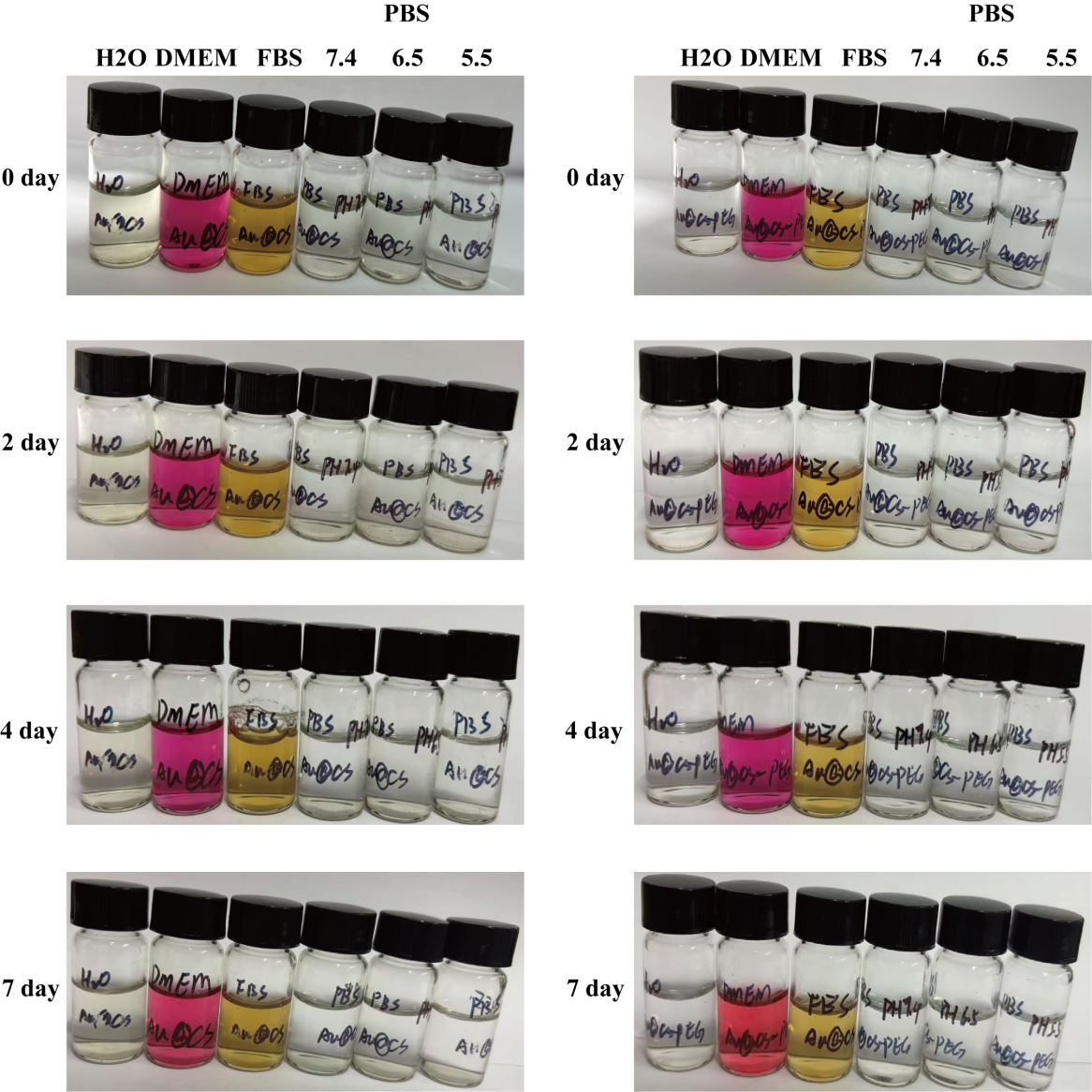
Figure S3.** Stability of Au@Cs and Au@Cs-HA-PEG nanoparticles in various solutions at Day 0, 2, 4, and 7.

**Figure S4.** Fourier transform infrared spectroscopy display
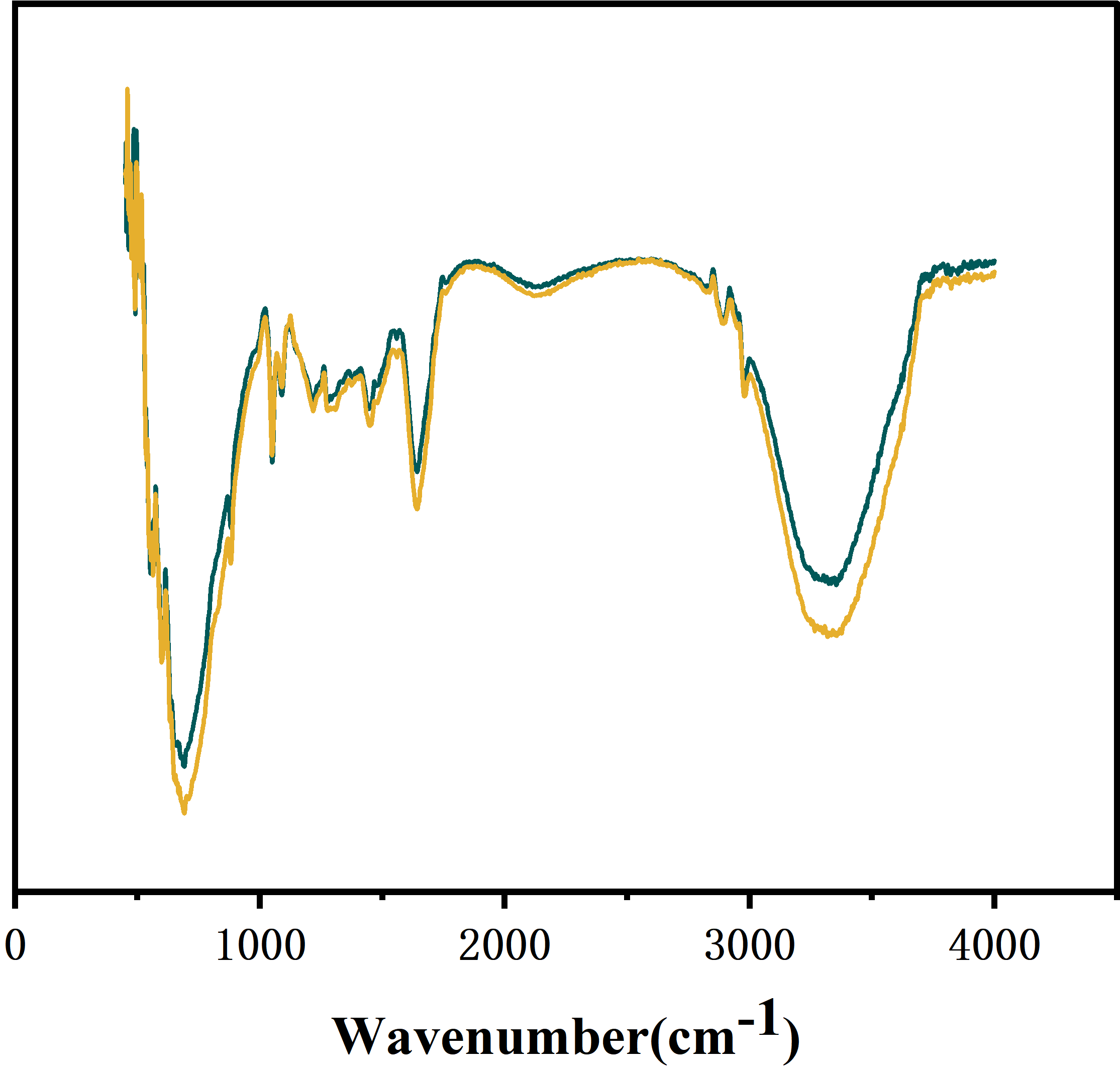
.


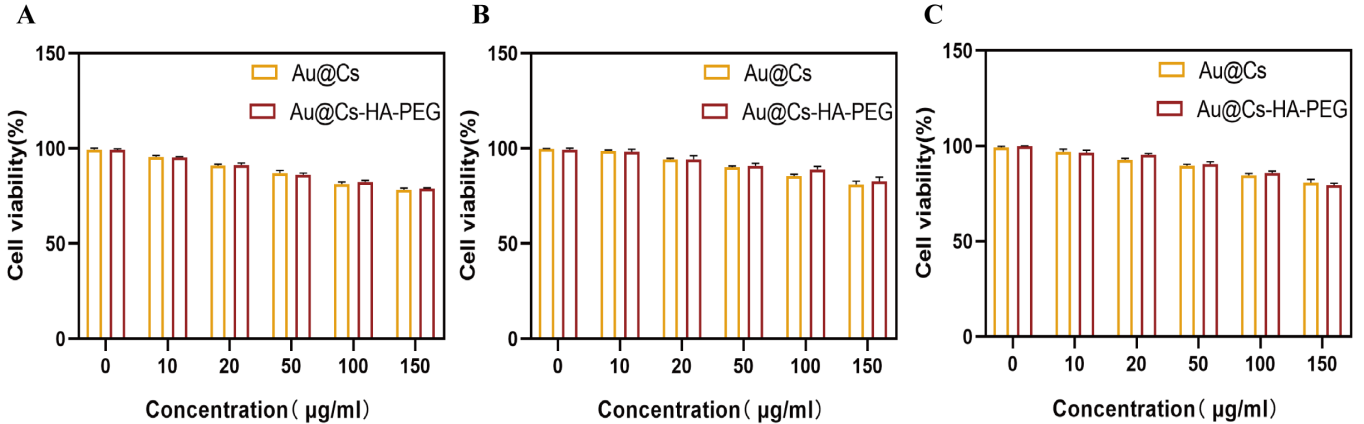


**Figure S5.** The viability of (A) 5Y5Y, (B) BV2 and (C) HT22 cells after incubation with different concentrations of Au@Cs-HA-PEG was evaluated by the CCK-8 method. Data are expressed as mean ± SEM.


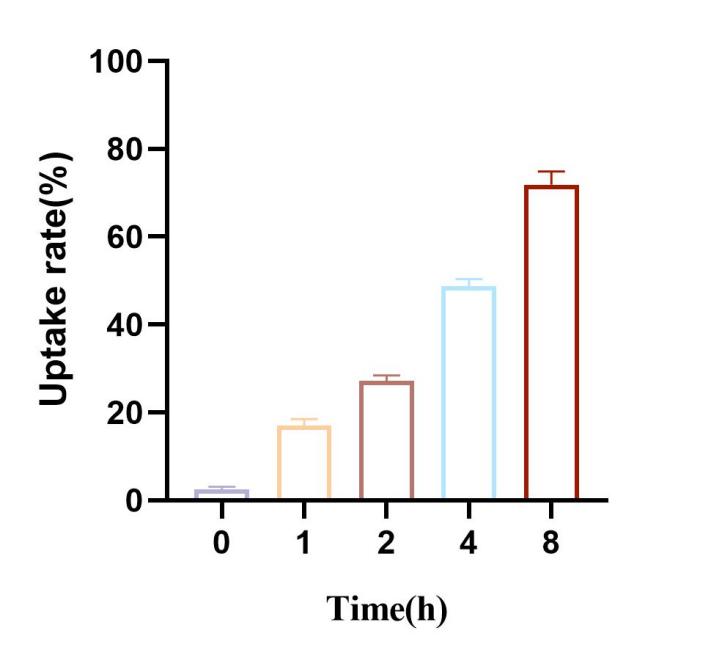

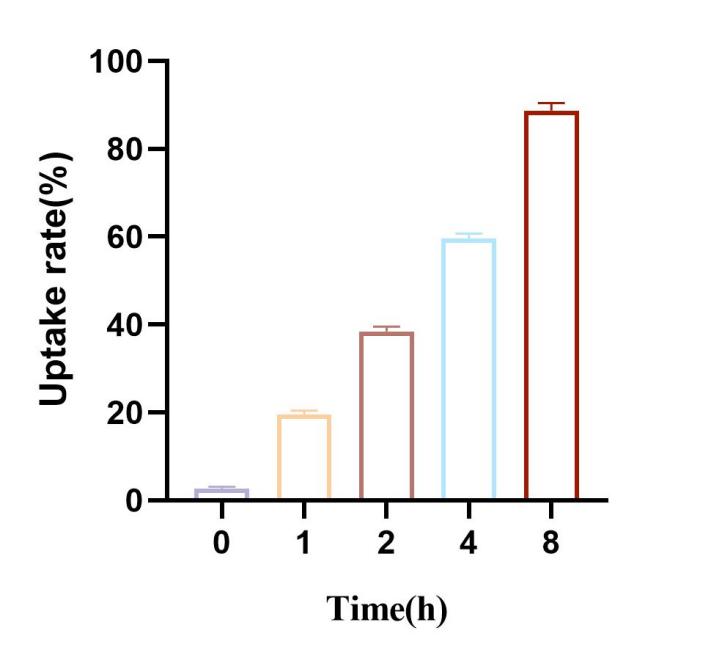


**Figure S6, 7.** Quantitative analyses of uptake in BV2 cells and HT22 cells. The data are expressed as mean ± SD.


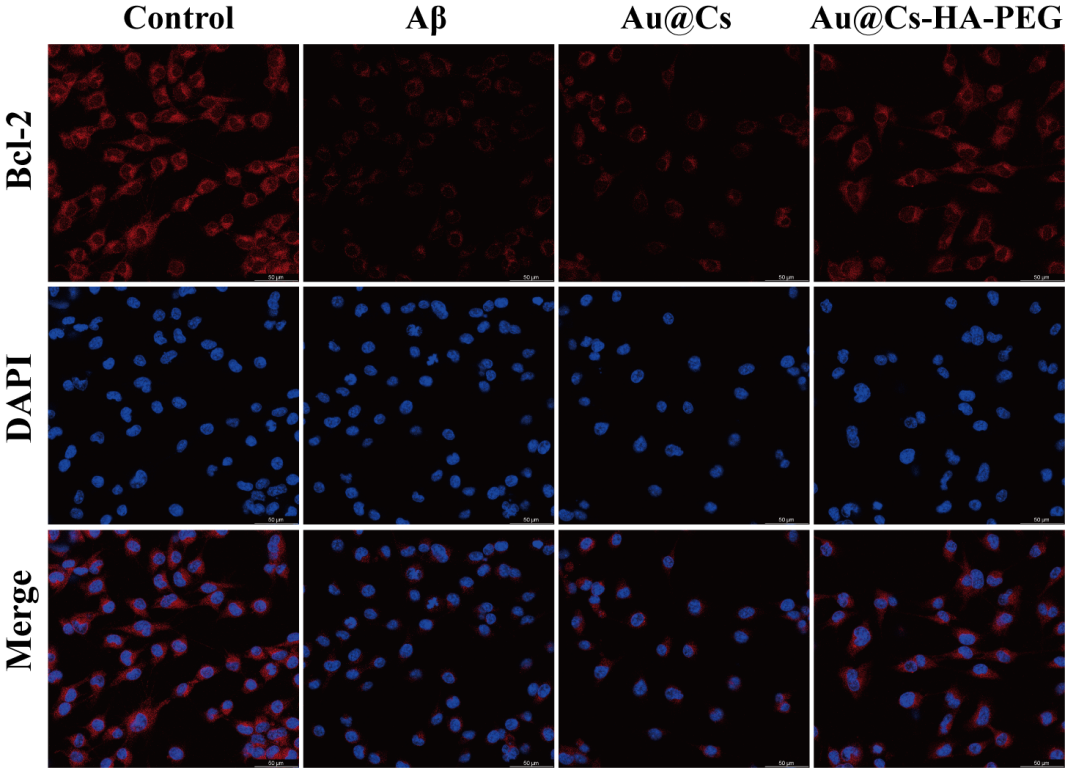


**Figure S8.** Representative confocal fluorescence images of Bcl-2 staining, red, Bcl-2 protein; blue, DAPI. Size: 50 μm.


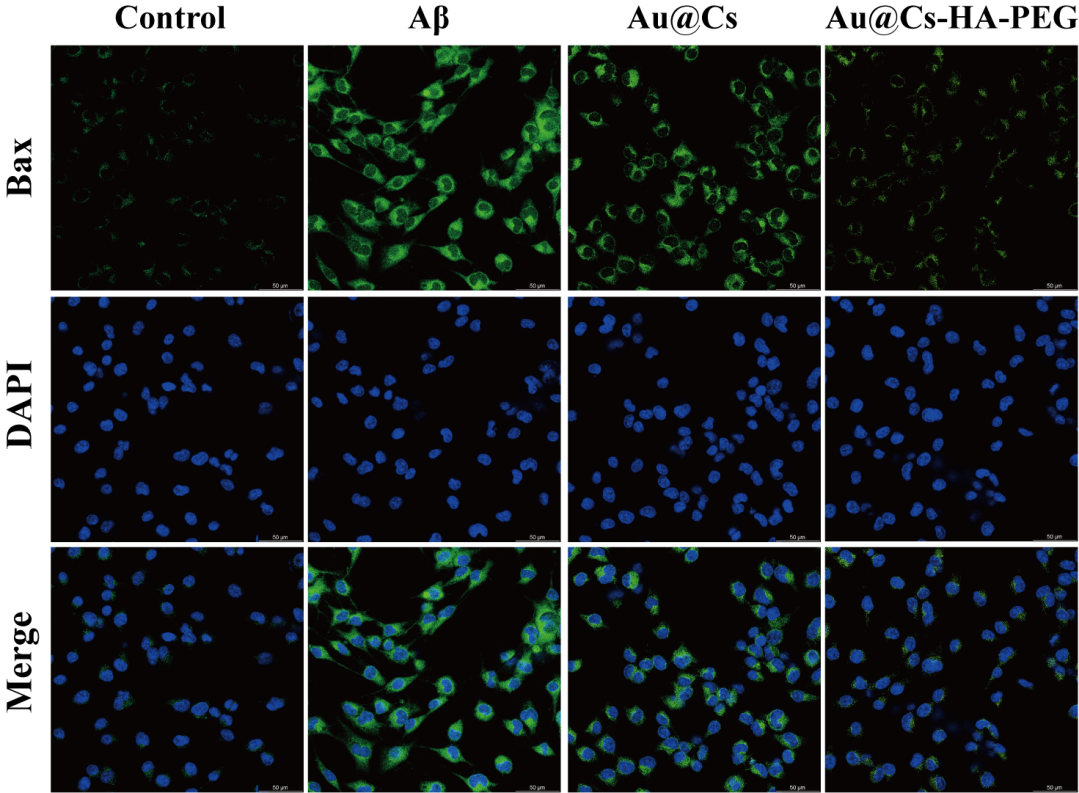


**Figure S9.** Representative confocal fluorescence images of Bax staining, red, Bax protein; blue, DAPI. Size: 50 μm.


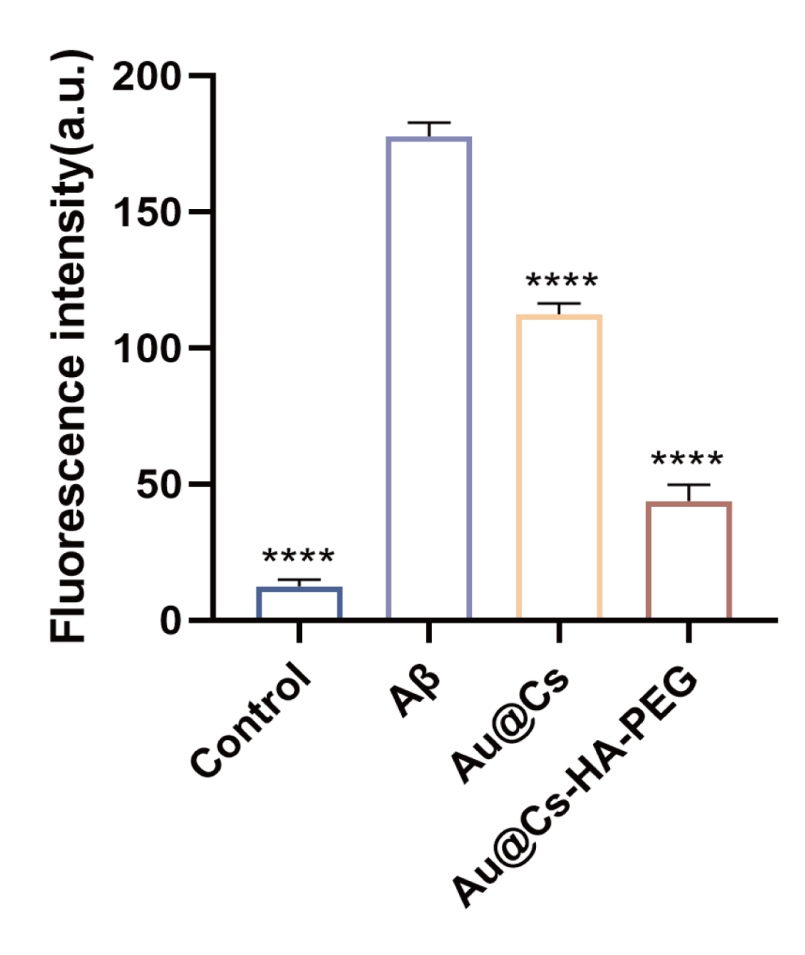


**Figure S10.** Quantitative analysis of THT fluorescence intensity of cells after treatment with different nanomedicines. Data are expressed as mean ± SEM (n = 3). *P < 0.05, **P < 0.01, ***P < 0.001, and ****P < 0.0001.


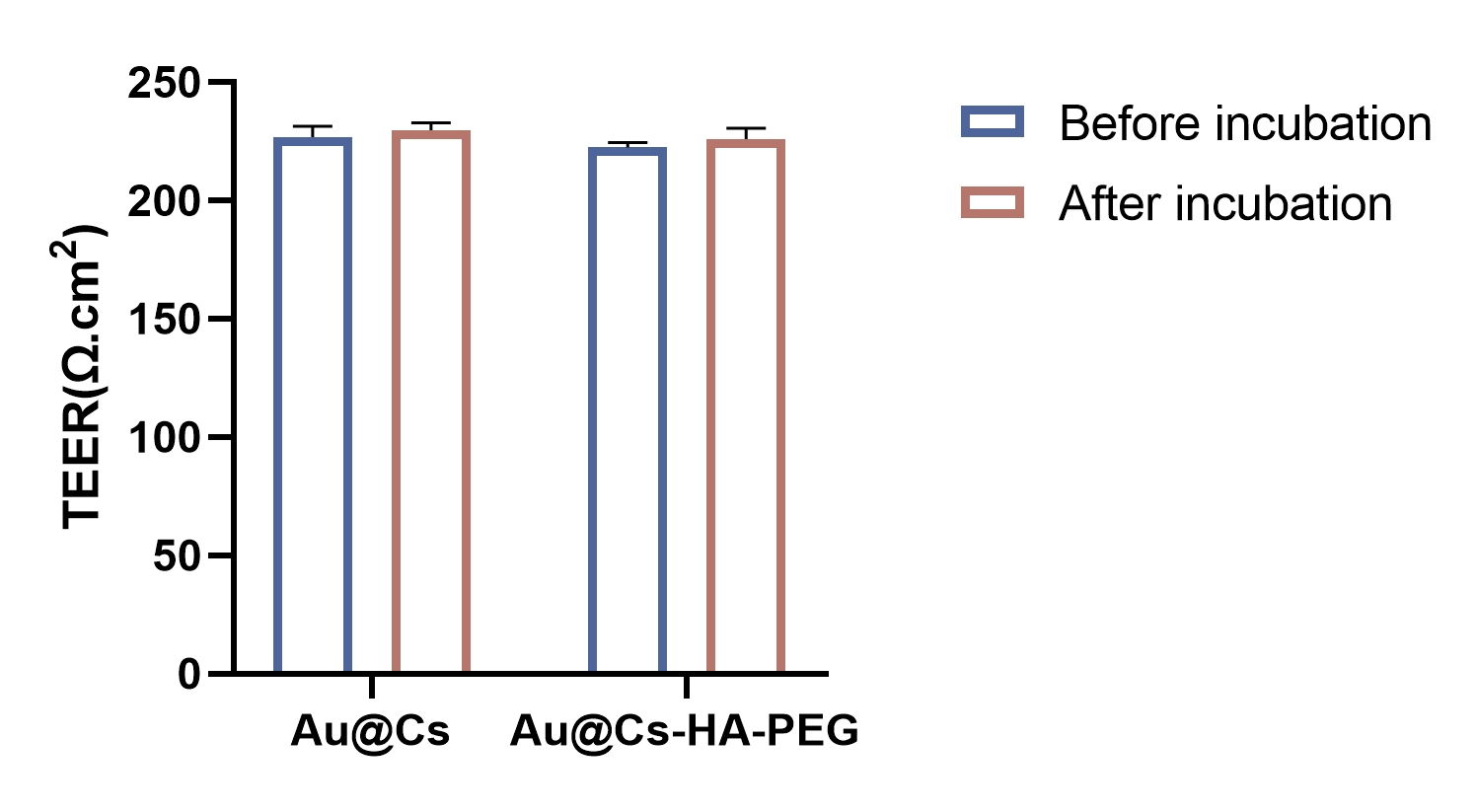


**Figure S11.** The TEER values of the bEnd.3 monolayer before and after incubation with nanoparticles (n = 3, mean ± SD).


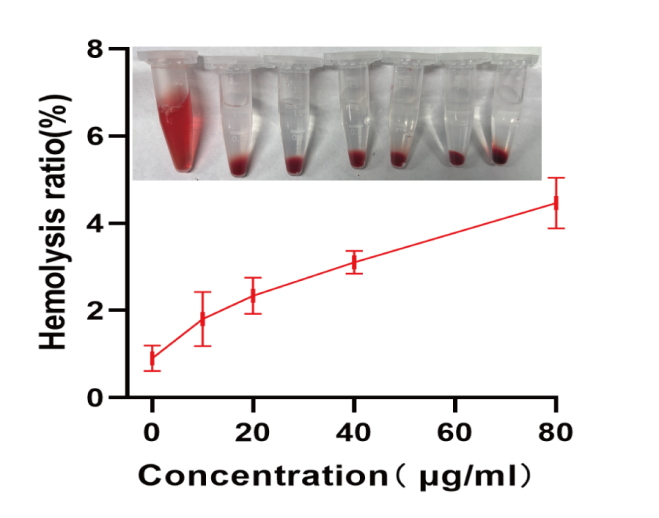


**Figure S12.** Hemolysis rate of various doses of Au@Cs-HA-PEG (n = 3, mean ± SD).


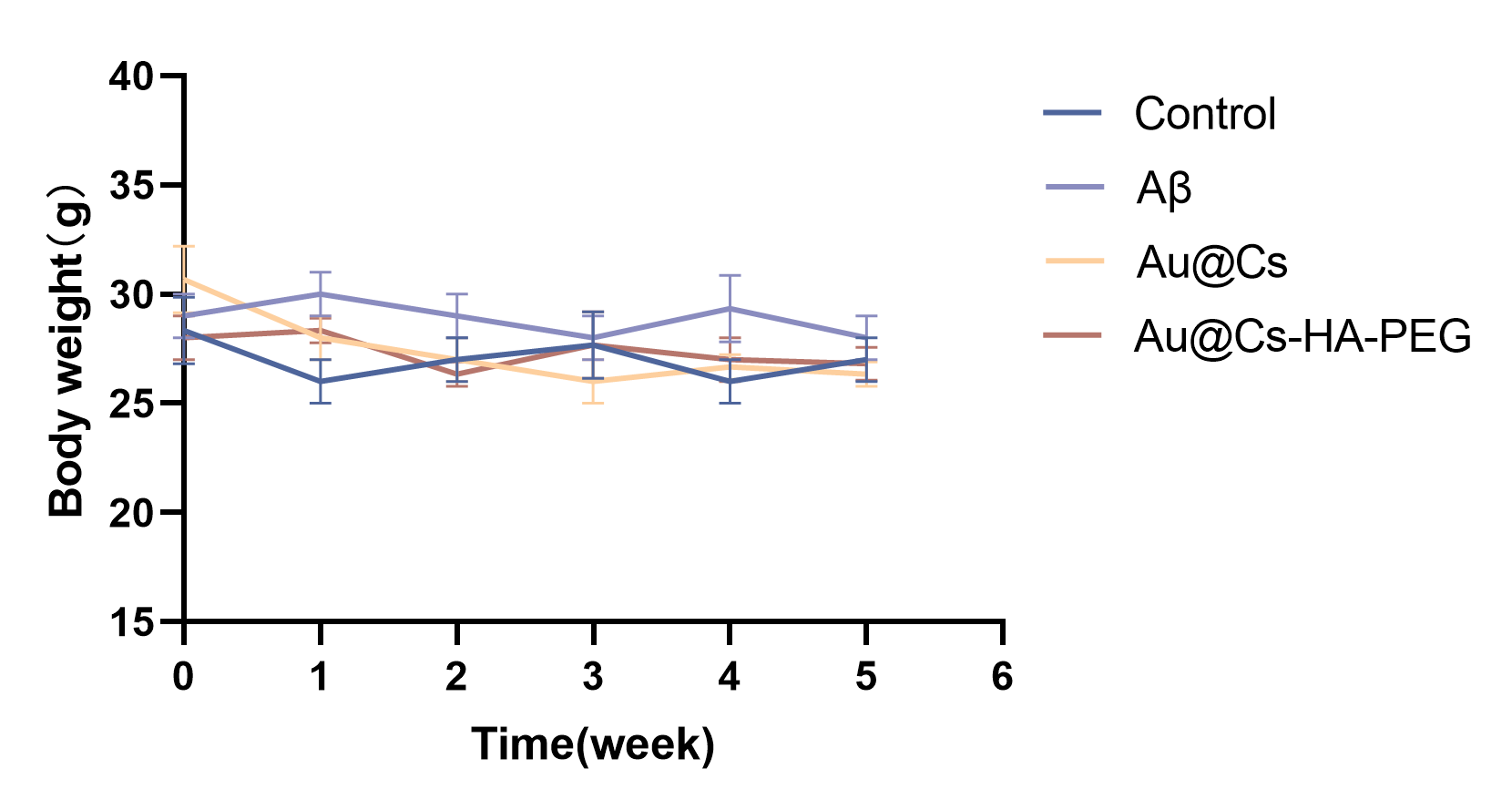


**Figure S13.** Body weight changes of mice during various treatments. Data are presented as mean ± SEM.


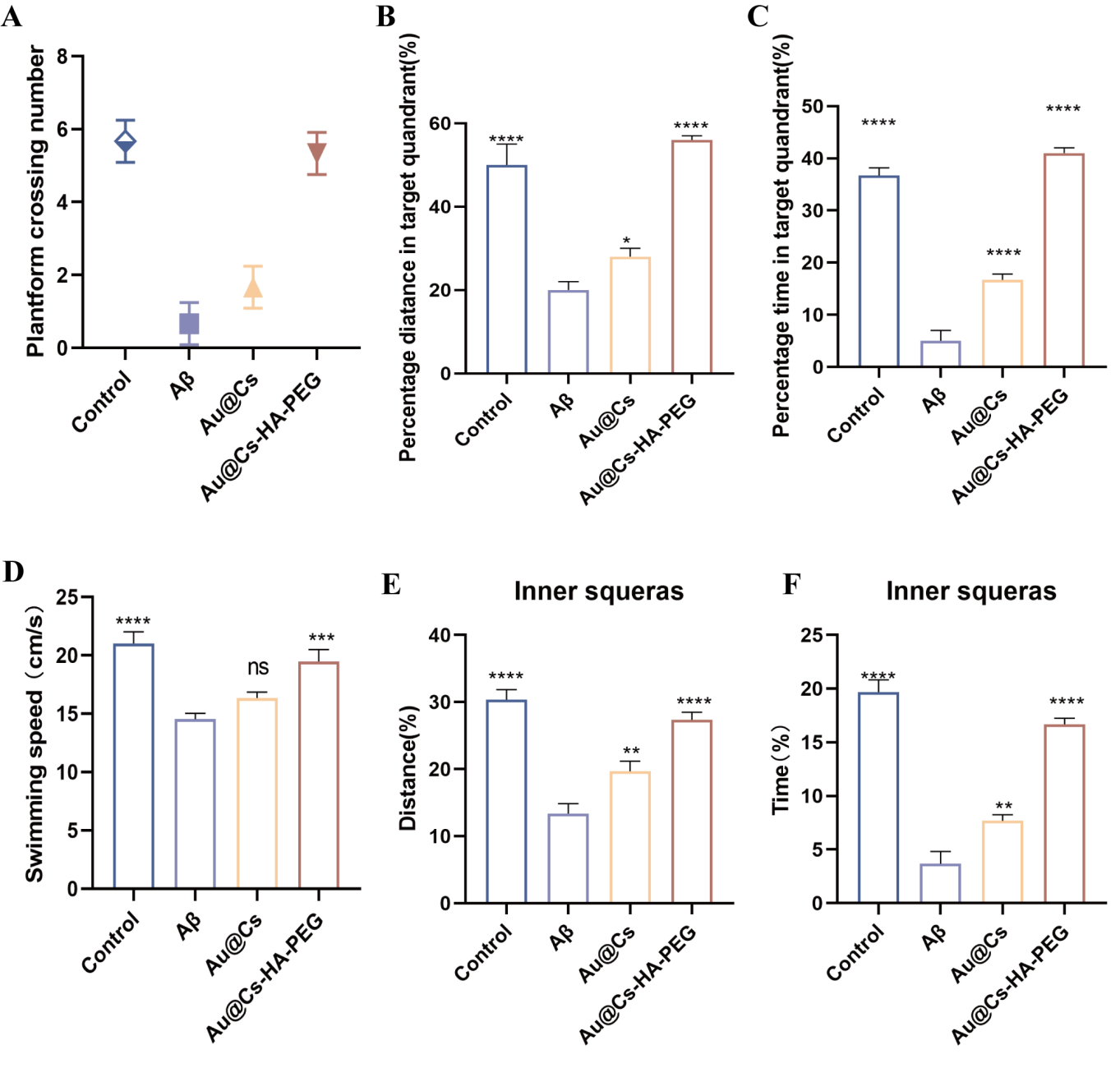


**Figure S14.** (A) The number of times the mice crossed the platform during the MWM experiment (B, C) Time and distance of the mice in the target quadrant. (D) Swimming speed of mice. (E, F) Time and distance of mice in the central region in open field experiments. Data are expressed as mean ± SEM. *P < 0.05, **P < 0.01, ***P < 0.001, and ****P < 0.0001.


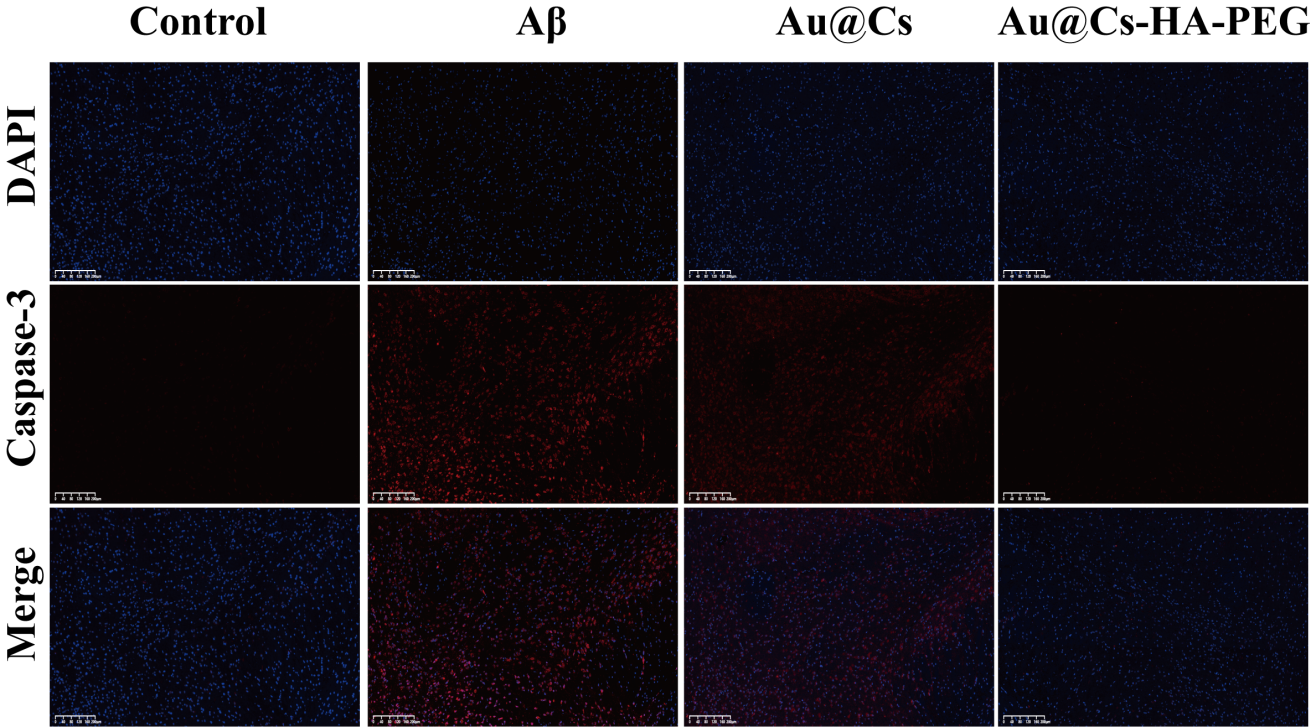


**Figure S15.** Immunofluorescence analysis of Caspase-3 in mouse brains treated with different nanomedicines.


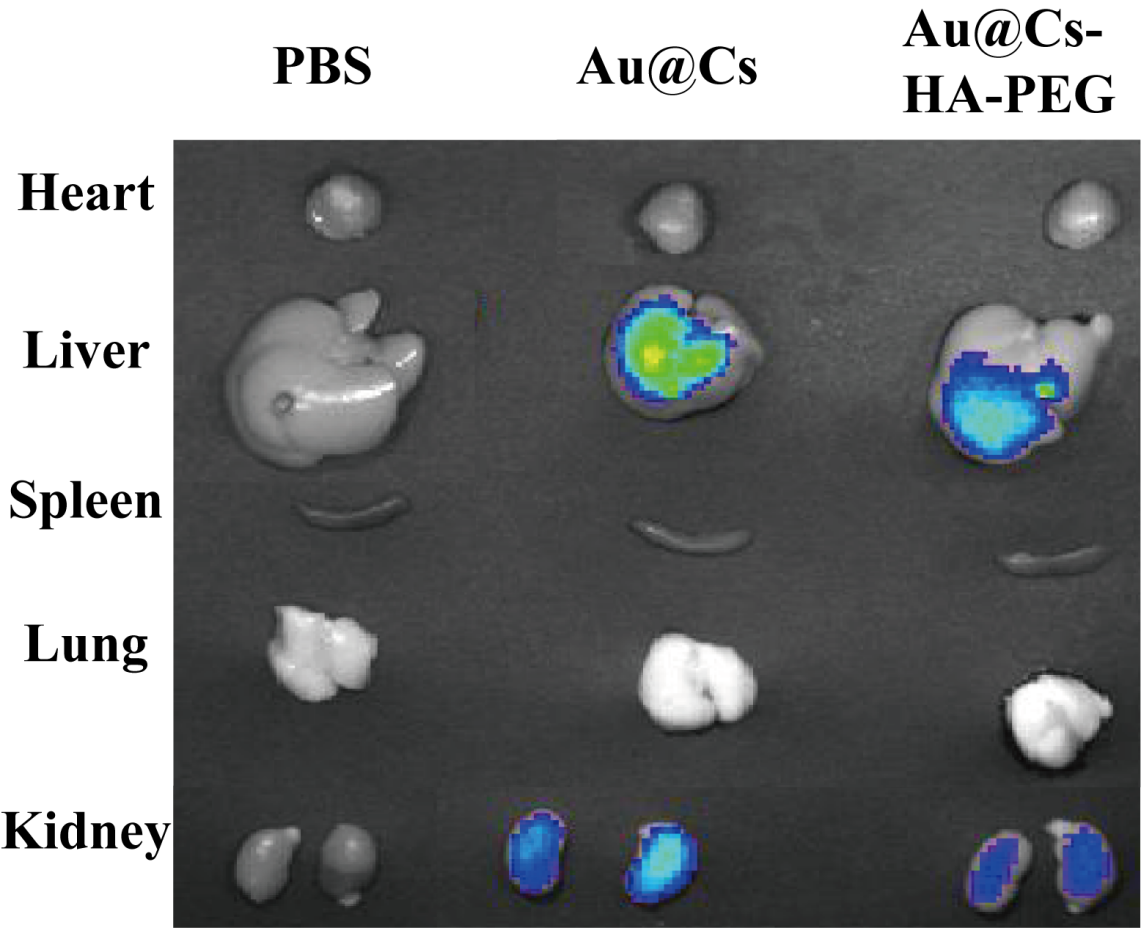


**Figure S16.** In vitro fluorescence images of major representative organs 24 h after administration. Data are expressed as mean ± SEM. *P < 0.05, **P < 0.01, ***P < 0.001, and ****P < 0.0001.

**References**

[1] H. Sun, Y. Zhong, X.D. Zhu, H.W. Liao, J.Y. Lee, Y. Chen, L.J. Ma, J.F. Ren, M. Zhao, M.J. Tu, F.Y. Li, H. Zhang, M. Tian, D.S. Ling, A Tauopathy-Homing and Autophagy-Activating Nanoassembly for Specific Clearance of Pathogenic Tau in Alzheimer's Disease, Acs Nano 15(3) (2021) 5263–5275 https://doi.org/10.1021/acsnano.0c10690.

[2] K. Qian, X.Y. Bao, Y.X. Li, P.Z. Wang, Q. Guo, P. Yang, S.T. Xu, F.Z. Yu, R. Meng, Y.L. Cheng, D.Y. Sheng, J.X. Cao, M.J. Xu, J. Wu, T.Y. Wang, Y.H. Wang, Q. Xie, W. Lu, Q.Z. Zhang, Cholinergic Neuron Targeting Nanosystem Delivering Hybrid Peptide for Combinatorial Mitochondrial Therapy in Alzheimer's Disease, Acs Nano 16(7) (2022) 11455–11472 https://doi.org/10.1021/acsnano.2c05795.

[3] K.Z. Ge, Y.F. Mu, M.Y. Liu, Z.T. Bai, Z. Liu, D.Q. Geng, F.L. Gao, Gold Nanorods with Spatial Separation of CeO<sub>2</sub> Deposition for Plasmonic-Enhanced Antioxidant Stress and Photothermal Therapy of Alzheimer's Disease, Acs Applied Materials & Interfaces 14(3) (2022) 3662–3674 https://doi.org/10.1021/acsami.1c17861.

[4] Y.N. Liu, D.J. Zhao, F. Yang, C.H. Ye, Z.Y. Chen, Y.H. Chen, X.M. Yu, J.Y. Xie, Y. Dou, J. Chang, <i>In Situ</i> Self-Assembled Phytopolyphenol-Coordinated Intelligent Nanotherapeutics for Multipronged Management of Ferroptosis-Driven Alzheimer's Disease, Acs Nano 18(11) (2024) 7890–7906 https://doi.org/10.1021/acsnano.3c09286.

[5] K. Ge, Z. Li, A. Wang, Z. Bai, X. Zhang, X. Zheng, Z. Liu, F. Gao, An NIR-Driven Upconversion/C(3)N(4)/CoP Photocatalyst for Efficient Hydrogen Production by Inhibiting Electron-Hole Pair Recombination for Alzheimer's Disease Therapy, ACS Nano 17(3) (2023) 2222–2234 https://doi.org/10.1021/acsnano.2c08499.

[6] P. Liu, T. Zhang, Y. Wu, Q. Chen, T. Sun, C. Jiang, A Peptide-Drug Conjugate-Based Nanoplatform for Immunometabolic Activation and In Situ Nerve Regeneration in Advanced-Stage Alzheimer's Disease, Adv Mater 36(46) (2024) e2408729 https://doi.org/10.1002/adma.202408729.

[7] Y. Hu, Y. Miao, Y. Zhang, X. Wang, X. Liu, W. Zhang, D. Deng, Co-Assembled Binary Polyphenol Natural Products for the Prevention and Treatment of Radiation-Induced Skin Injury, ACS Nano 18(40) (2024) 27557–27569 https://doi.org/10.1021/acsnano.4c08508.
